# Reconstruction of feeding behaviour and diet in Devonian ctenacanth chondrichthyans using dental microwear texture and finite element analyses

**DOI:** 10.1098/rsos.240936

**Published:** 2025-01-29

**Authors:** Merle Greif, Ivan Calandra, Stephan Lautenschlager, Thomas M. Kaiser, Mohammed Mezane, Christian Klug

**Affiliations:** ^1^ Department of Palaeontology, University of Zurich, Karl-Schmid-Strasse 4, Zurich 8006, Switzerland; ^2^ Imaging Platform at LEIZA (IMPALA), and Laboratory for Traceology and Controlled Experiments (TraCEr), MONREPOS Archaeological Research Centre, Leibniz-Zentrum für Archäologie, Neuwied 56567, Germany; ^3^ School of Geography, Earth and Environmental Sciences, Lapworth Museum of Geology, University of Birmingham, Edgbaston, Birmingham, UK; ^4^ Centre for Taxonomy and Morphology, Section Mammalogy and Paleoanthropology, Leibniz Institute for the Analysis of Biodiversity Change (LIB), Martin-Luther-King-Platz 3, Hamburg 20146, Germany; ^5^ Merzouga Errachidia Province, Merzouga BO 520202, Morocco

**Keywords:** Devonian, DMTA, chondrichthyans, feeding behaviour, tooth wear, FEA

## Abstract

Devonian ctenacanth chondrichthyans reached body sizes similar to modern great white sharks and therefore might have been apex predators of the Devonian seas. However, very little is known about the diet and feeding behaviours of these large ancestral sharks. To reconstruct their ecological properties, teeth of the large Famennian (Late Devonian) chondrichthyan *Ctenacanthus concinnus* from the Anti-Atlas, Morocco, were analysed. The teeth show strong tooth wear with deep horizontal as well as vertical scratches. Dental microwear texture analysis, a well-established method for the reconstruction of diet and commonly used in terrestrial vertebrates, was applied for the first time, to our knowledge, to Palaeozoic vertebrates in this study. Furthermore, finite element analysis was performed to test the biomechanical properties of the teeth. By combining both analyses, as well as palaeoenvironmental data and tooth morphology, we demonstrate that the results from only one method can be insufficient and misleading. *Ctenacanthus concinnus* most likely was an opportunistic feeder like many modern sharks. Direct evidence and the results of our analyses suggest that *Ctenacanthus* fed on ectocochleate cephalopods, other chondrichthyans and further vertebrates using a combination of head movements including lateral head shaking to cut large prey items.

## 1. Introduction

The dentitions of fossil and modern cartilaginous fishes (sharks, rays and holocephalans) show great morphological and functional disparity [1–3]. Several groups, including many rays and holocephalans, evolved tooth plates for crushing hard-shelled prey [4–6]. Planktivorous chondrichthyans such as manta rays, whale sharks and basking sharks, usually have countless, proportionally minute teeth. Many modern sharks are known as dietary opportunists, often feeding on teleost fishes but also invertebrates such as crustaceans or cephalopods [7]. In these sharks a variety of behaviours for capturing prey are known, such as ram feeding, suction feeding, biting, filter feeding or combinations of these [7,8].

While in modern sharks, diets and feeding habits can be observed directly [7,9–16], in fossil chondrichthyans, direct evidence is scarce. In some rare cases, stomach contents or bromalites (digestive system remains [17,18]) are preserved and provide information about prey [19–22]. Indirect information regarding diet and feeding mechanisms of fossil chondrichthyans can be obtained from coprolites (fossilized faeces [17,18,23]), tooth marks in larger prey items [24,25], tooth morphology [26–28] or tooth wear [16,29–31]. However, the study of tooth morphology has led to conflicting results regarding feeding mechanisms and diet [26,27,29,32]. Heterodonty in shark dentitions (different tooth morphologies in a jaw) can hamper such studies [33,34], especially when the degree of heterodonty is unknown. Heterodonty is common in modern sharks (Elasmobranchs) and can be expressed in different ways: varying tooth shapes along the jaw or between teeth of the lower and upper jaw [34]. However, the correlation between tooth morphology and feeding strategies is still unclear and needs further investigation. For instance, Cooper *et al.* [27] concluded that dental characters are useful proxies for feeding mechanisms as well as non-ingesta-related traits and that these could be useful for reconstructing ecological traits of fossil sharks. However, a connection between shark tooth morphology and function has not yet been confirmed by biomechanical investigation [26]. Furthermore, tooth wear analyses have been used to assess the functional implications of tooth morphology [16,29]. However, these studies are limited in number and led to conflicting results [16,29–31] even though recent studies show that tooth wear can provide valuable information [29]. However, to date, there is no agreed standard available against which to compare and analyse tooth wear patterns in chondrichthyans through time.

Dental wear is produced by consumption of food items and by other ingesta, such as fine sediment or grit, that all leave scratch marks and pits on the enamel [35] (or here enameloid [36]) layer of the tooth surface. Tooth wear can additionally be produced by attrition (loss of tooth structure owing to tooth–tooth contact) and abfraction (microstructural loss of dentine in stressed areas) [37]. In sharks, teeth are arranged in a ‘conveyor belt’ like manner including functional and replacement teeth. After internal development at the dental lamina, teeth erupt into the mouth and migrate into a functional position [38,39]. The teeth stay functional, i.e. are used during feeding, for a certain duration until the worn teeth are replaced by the next tooth in each tooth file [40]. The longer an individual tooth remains functional, the more intense the tooth wear will probably be. Tooth replacement in modern sharks is comparatively fast and teeth are replaced within only a few weeks or even days [38,41–44]. Fast tooth replacement results in less ingesta-to-tooth contact and minimal tooth wear. By contrast, early cladodont chondrichthyans are thought to have retained their teeth throughout their life instead of shedding and replacing them [45,46]. Tooth retention appears to be a basal feature in early chondrichthyans, which had to be modified to establish a functional tooth battery of sharp, serrated or slender pointed teeth [44].

**Figure 1.**
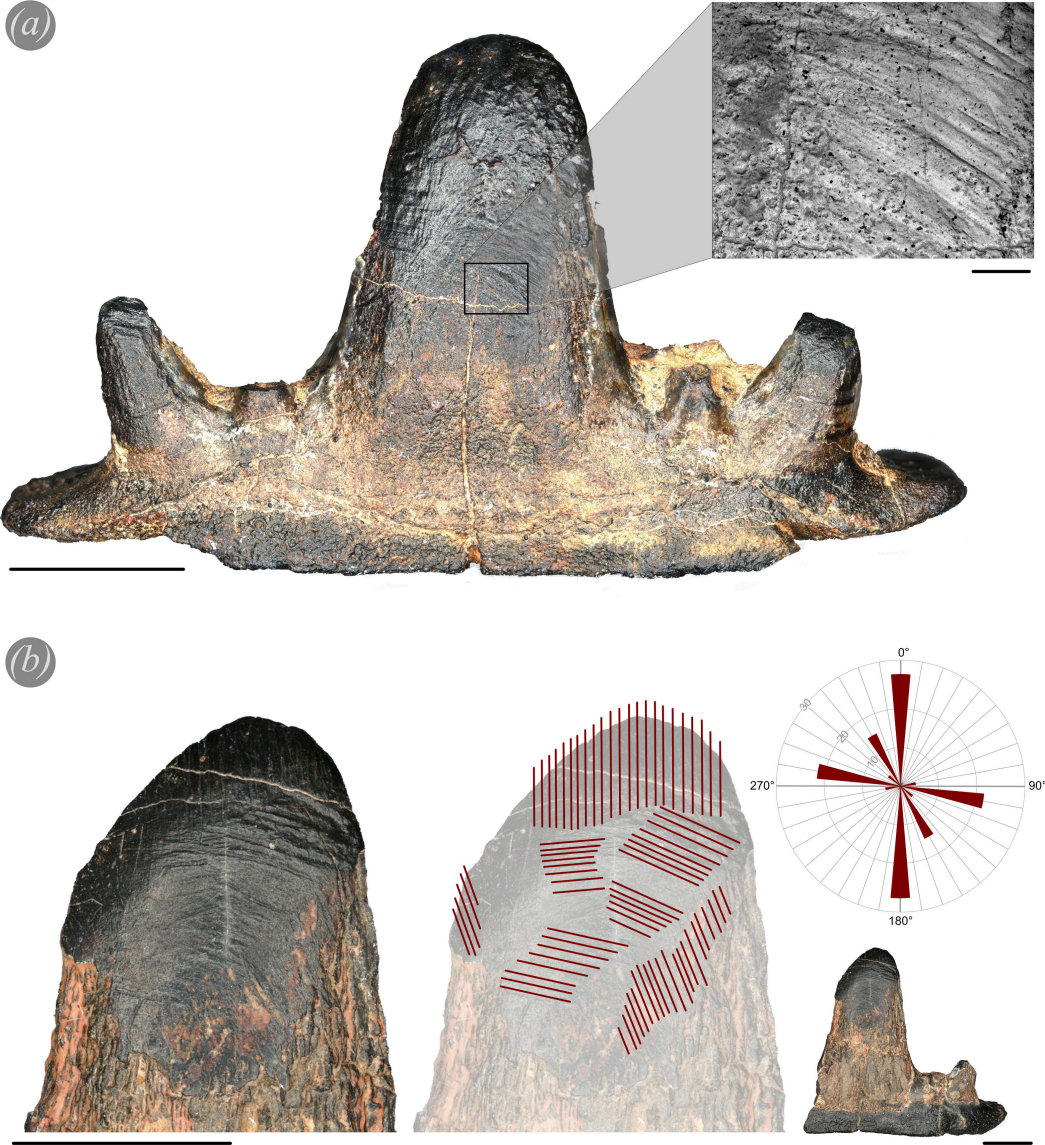
(*a*) PIMUZ A/I 5253 (tooth A, all teeth in electronic supplementary material, figures S1 and S2) and detailed scanning electron microscope image (scale bar = 500 μm) showing the wear in the abapical part of the wear facet. The tooth shows a highly worn wear facet and strong wear in the outermost cusplets, mainly visible on the left cusplet. (*b*) Wear facet of PIMUZ A/I 5254 (tooth B, all teeth in electronic supplementary material, figures S1 and S2) as well as an overview picture of tooth B. Red lines represent main scratches and their directions. A rose diagram (*x*-axis = scratch angle; *y*-axis = number of scratches) of all counted scratches shows that there are two main directions: horizontal and vertical. The vertical scratches are mainly in the apical part of the wear facet while the horizontal scratches are more in the middle to abapical parts of the wear facet. Scale bars 5 mm.

The early chondrichthyan genus *Ctenacanthus*, first described by Agassiz [47], possessed large cladodont teeth. It is known from strata ranging from the Famennian (Late Devonian) to the Permian. Based on isolated elements, it was the largest chondrichthyan of the Devonian worldwide, and it was not before the Carboniferous that similarly large chondrichthyans such as some other ctenacanthiforms [48,49] and edestids (holocephalans) [50] evolved. Many teeth of *Ctenacanthus concinnus* show intense tooth wear and wear facets (figure 1), to a degree not seen in any other chondrichthyan teeth. Such wear patterns, with the abundant and sometimes deep scratches as well as wear facets, may have been caused by a preference for hard-shelled or bony prey. Here, we test this hypothesis by investigating the teeth of the large chondrichthyan *Ct. concinnus* as well as teeth of the related species *Ctenacanthus tumidus* from Morocco. Both species were already reported from Morocco [51,52] and are also known from the Cleveland shale in Ohio, USA ([33,53]; see also [2, fig. 65] for *Ct. tumidus*). We use a combination of dental microwear texture analysis (DMTA) and finite element analysis (FEA). Torices *et al.* [54] formerly used this approach to reconstruct the feeding behaviours of coelurosaurian dinosaurs and provide information on the material properties of consumed food items (DMTA) as well as the biomechanical capabilities of the teeth (FEA).

Furthermore, we investigate whether the diet of Palaeozoic cartilaginous fishes can be reconstructed in the absence of direct evidence such as stomach or gut contents using tooth wear, as well as morphological, biomechanical and environmental information. Using Devonian chondrichthyan teeth that show intense wear, we examine whether tooth wear can give reliable information on diet and feeding mechanisms.

## 2. Material

The teeth of *Ct. concinnus* used in this study were collected from the Famennian (379−359 Myr ago, [55]) *Gonioclymenia* limestone of the Moroccan Anti-Atlas. The examined teeth are from the Tafilalt region, northwest of Taouz. The Tafilalt represents a moderately shallow marine basin that was separated from the neighbouring Maїder Basin by a pelagic platform in the Devonian [56,57]. This region is renowned for its early vertebrate fossils, predominantly chondrichthyans and placoderms, in considerably large numbers and sometimes exceptional preservation [22,51,58–65].

For DMTA, nine *Ct. concinnus* teeth were studied. Seven of these (PIMUZ A/I 5253-PIMUZ A/I 5259, denoted A–G; electronic supplementary material, S1) were found associated with each other, although in the scree, at 30.99598° N, 4.05670° W (Rich Tamirant). These teeth therefore most likely belonged to one individual. Two additional teeth (PIMUZ A/I 5260 and PIMUZ A/I 5261, denoted H and I; electronic supplementary material, figures S1 and S2) were found together with specimen PIMUZ A/I 5262 at 31.01460° N, 4.06534° W. One additional tooth of the same specimen (PIMUZ A/I 5264, tooth J) was computed tomography (CT)-scanned, and the resulting three-dimensional model was used for FEA. For comparative reasons, teeth of *Ct. tumidus* from the Middle Famennian Maїder Basin, Morocco, were included in this study (electronic supplementary material, S1; description *Ct. tumidus*). These teeth are preserved in the matrix but are disarticulated. The teeth of *Ct. tumidus* (PIMUZ A/I 5263) were found at Madene El Mrakib 30.45056° N, 4.42829° W.

**Figure 2.**
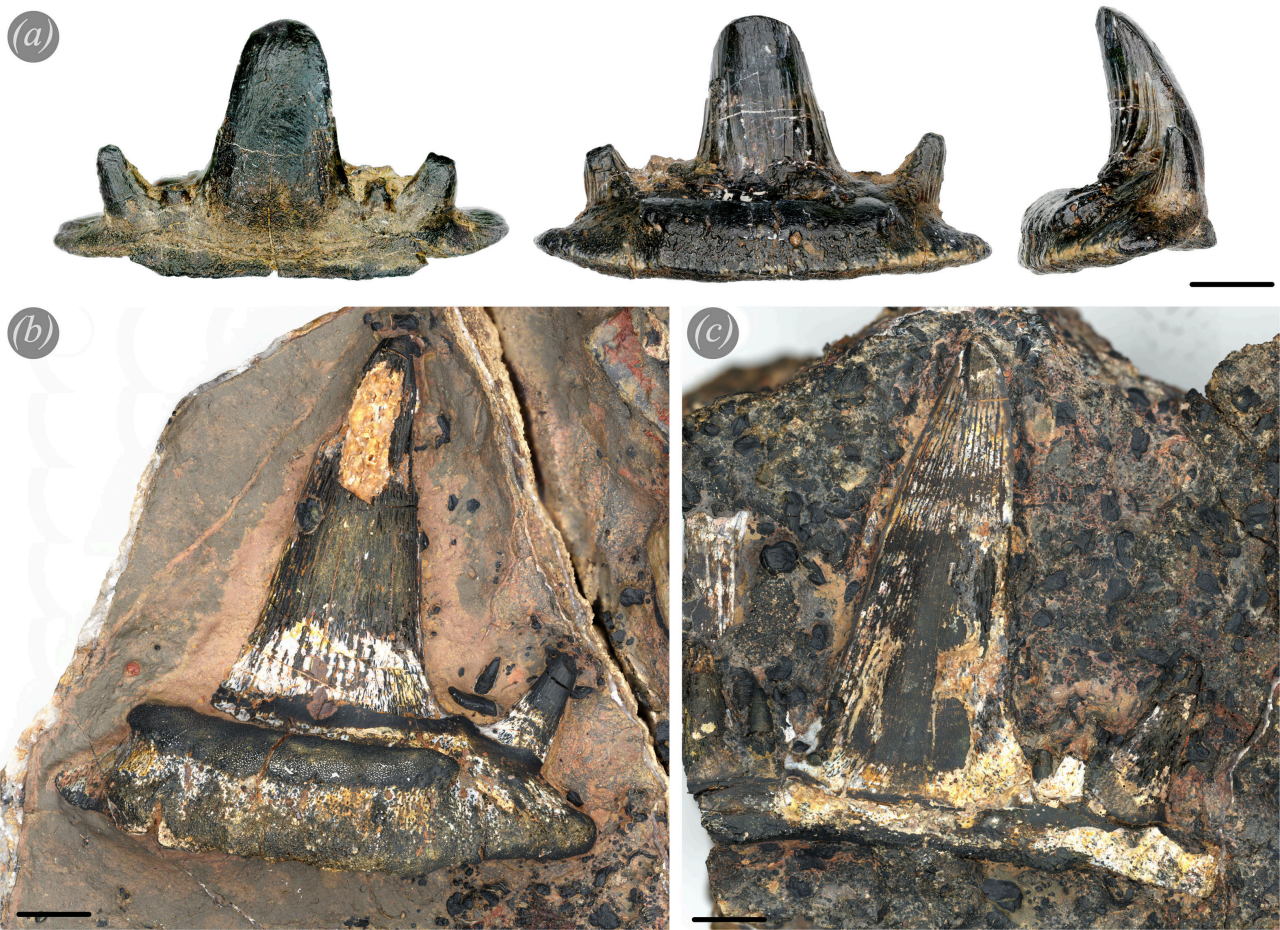
Overview of *Ctenacanthus concinnus* teeth. (*a*) PIMUZ A/I 5253 from labial, lingual, mesial view (left to right). (*b*) PIMUZ A/I 5262 tooth embedded in matrix in lingual view. The tooth shows the originally pointed apex with little sign of apical wear. (*c*) PIMUZ A/I 5262 tooth in matrix and surrounded by scales, from the labial side showing the apex and three cusplets on each side. Scale bars 5 mm.

Further ctenacanthiform specimens from Morocco preserve teeth as well. These are normally surrounded by matrix and could not be prepared without the risk of damaging the tooth surface making it impossible to distinguish preparation damage from tooth wear. We therefore focused on the isolated teeth of *Ct. concinnus* that preserve large wear facets (figures 1 and 2). In these specimens, the tooth wear resulted in a heavily worn, blunt apex in contrast to the originally sharp tip. Some teeth are even worn down almost to the base (electronic supplementary material, figure S1G). We exclude taphonomical processes as the cause of the high degree of wear because it is only apparent in the apical region of the teeth rather than on all tooth regions. Wear facets that show mesowear and microwear can be over 5 mm wide in some cases and thus are visible to the naked eye on the labial side extending from the uppermost part approximately one-quarter down the tooth. In some teeth, strong signs of wear are also visible in the outermost cusplets.

3. Methods

### 3.1. Microscopic documentation

All teeth were photographed using a Keyence digital microscope VHX-7000 with a VHX-7100 fully integrated head, and two lens system (VHX-E20, VHX-E100). The images were adjusted in colour and contrast using Adobe Photoshop CS6 and Affinity Designer/Photo. Scanning electron microscope (SEM) pictures were taken at the Centre for Microscopy and image analysis at the University of Zurich using a Jeol JSM-IT700HR field emission SEM. SEM pictures were acquired in low vacuum (30 Pa) using the secondary electron detector (all original SEM images and their metadata are available on Zenodo: https://doi.org/10.5281/zenodo.14605615).

The wear facets of PIMUZ A/I 5253-PIMUZ A/I 5259 (teeth A–G) were first observed using a stereo-microscope Leica MZ16 F, in an attempt to record the number of scratches, as well as their directions, and subsequently analyse the wear patterns (electronic supplementary material, figure S3). Since this method is highly subjective and a high error is to be expected, we decided to analyse tooth wear using DMTA.

### 3.2. Data acquisition for dental microwear texture analysis

DMTA is a well-established method used in many extant as well as extinct vertebrate groups such as terrestrial mammals [66–74], including hominoids [75–77], and recently also marine mammals [37,78–84] and reptiles [85,86], including dinosaurs [87–90]. To date, in chondrichthyans, DMTA has so far only been applied a very few times [16,31]. Yet, the method is beneficial in a number of ways [31] and can detect differences between individuals and populations with small sample sizes [27,65,66].

Topographical height maps of the wear facets (commonly termed scans) of nine teeth of *Ct. concinnus* were acquired with a high-resolution confocal microscope (Sensofar Plu S-Neox, LIB Hamburg) using the confocal mode and blue LED light. Each tooth was oriented with the labial side facing dorsally and the apex pointing straight away from the observer to allow the directions of scratches to be recorded. For each tooth, two to four measurements were taken at non-overlapping positions (electronic supplementary material, figure S4) prioritizing areas that show intense wear. Macrowear scratches originate from single events and have no influence on microwear. At every position, three measurements were repeated with the 20× (Nikon TU Plan ELWD Fluor 20×/0.45) and 100× (Nikon TU Plan ELWD 100×/0.8) objectives to average out measurement noise by calculating the mean height map (following Calandra [91]). Unfortunately, the ‘shift’ operator in Mountains (Digital Surf), the post-processing software for DMTA scans employed, does not align the individual height maps properly, meaning that extracting the mean surface was not possible. Therefore, the three measurements at each position were used individually instead of the mean height map and we were not able to average out the measurement noise. With the 20× objective, an area of 877.20 µm × 660.48 µm was measured and with the 100× objective an area of 175.44 µm × 132.10 µm. Each height map includes 1360 x 1024 pixels (0.65 µm pixel^−1^ using the 20× objective and 0.13 µm pixel^−1^ using the 100× objective). The outputs were saved as *.*plux* files. The measured surfaces were processed using the software Mountains following the workflow by Calandra [91]. The ratios of non-measured points (NMP, in %), a measure of data quality, were split into three categories: <10% (good), ≥10% and <20% (intermediate) and ≥20% (bad). Descriptive statistics are computed separately for each category. Height maps with more than 20% (NMP) were excluded from the plots; the other two categories are shown with different symbols for transparency. Results for the intermediate category should be treated with caution, even though the same pattern is visible from both categories (see below). Thirty-five parameters from five types of analysis were calculated: International Organization for Standardization (ISO) 25178-2, scale-sensitive fractal analysis [92,93], furrow analysis, texture isotropy and texture direction (electronic supplementary material, table S1). Mountains documents are available on Zenodo: https://doi.org/10.5281/zenodo.14605615 in both MNT and PDF formats. The most common surface parameters of all 35 parameters analysed for dietary reconstructions are *epLsar* and *Asfc. epLsar* describes the anisotropy of scratches in the wear facets and can generally be explained by the toughness of food or ingesta that caused wear during sliding contacts. *Asfc* describes the complexity of the wear surfaces and correlates positively with the hardness of food items or ingesta. All surface parameters are explained in the electronic supplementary material, table S1.

### 3.3. Statistical analysis

We tested for differences between all teeth of the two *Ct. concinnus* specimens, as well as for differences between the positions of the DMTs (dental microwear textures) on the wear facet of each tooth (apical and abapical, i.e. top and bottom measurements, electronic supplementary material, figure S4). Owing to the small number of samples, only descriptive statistics, biplots and principal component analysis (PCA) were performed.

Preparation of the data as well as all descriptive analyses were performed using R through RStudio. A research compendium containing raw data (result of the DMTA), all details of the data analysis (scripts), results (plots and descriptive statistics), software and package versions and citations can be found as HTML reports in the GitHub repository (https://github.com/ivan-paleo/DMTA.Ctenacanths) archived on Zenodo: https://doi.org/10.5281/zenodo.14604218.

### 3.4. Data acquisition for finite element analysis

FEA has become a widely used method to reconstruct biomechanical properties in vertebrate palaeontology [94–98]. External mechanical loads are computationally applied to a mesh (three-dimensional model), which is divided into a finite number of elements that resemble the object of interest [99]. Here, we apply loads from different directions to the teeth of *Ct. concinnus* to model different prey capture handling scenarios and compare them to modern analogues.

Specimen PIMUZ A/I 5264 (tooth J; electronic supplementary material, figure S1) was CT-scanned with a Nikon XT H 225 ST tomography scanner at the University of Zurich with the following parameters: 200 kV, voxel sizes in mm: *x*, *y* and *z*-axes = 0.052 mm; total slices: 2000; 16-bit images. Segmentation and three-dimensional reconstruction were performed using Mimics v. 19 (https://www.materialise.com/en/medical/software/mimics, Materialise, Leuven, Belgium) and Dragonfly v. 2022.2. Additionally, three teeth of modern shark species—*Prionace glauca* (blue shark, ID: 7-759/RZ, upper jaw), *Chlamydoselachus anguineus* (frilled shark, ID: 11-06/RZ, position in jaw cannot be determined) *and Hemipristis elongata* (snaggletooth shark, ID: 7-795/RZ, upper jaw)—from the private collection of René Kindlimann (Aathal) were CT-scanned using an industrial CT-scanner at Qualitech in Mägenwil, Switzerland, with the following parameters: *P. glauca*: 210 kV, 0.35 µA; voxel sizes in mm: *x*, *y* and *z*-axes = 0.0357 mm; total slices: 541; 16-bit images; *Ch. anguineus:* 210 kV, 0.35 µA; voxel sizes in mm: *x*, *y* and *z*-axes = 0.0357 mm; total slices, 132; 16-bit images. The teeth of *P. glauca* and *Ch. anguineus* were found isolated. The tooth of *H. elongata* was scanned together with the entire jaw and later isolated in Dragonfly v. 2022.2. The parameters of the scan for the jaw are the following: 210 kV, 0.35 µA; voxel sizes in mm: *x*, *y* and *z*-axes = 0.0738 mm; total slices: 901; 16-bit images.

For FEA, each model was imported as an STL surface mesh into Hypermesh (v. 11, Altair Engineering) for meshing and defining the boundary conditions. STL files of all teeth are available on Zenodo: https://doi.org/10.5281/zenodo.14605615. All models were scaled to the same surface area and assigned material properties for dentine (Young’s modulus *E* = 28 440 MPa, Poisson’s ratio *ν* = 0.30) [46]. No differentiation of the tooth material types was attempted as the scanned models are not of sufficient resolution to distinguish tissues. According to Herbst *et al*. [94] a differentiation of dental material does not affect the results of FEA. Simplifying the internal structures of the compared teeth in this study is therefore reasonable. Given the comparative approach used here, it is assumed that the effects of internal tissue properties would be consistent across all models [100,101].

For the application of realistic constraints, each tooth model was fixed along all degrees of freedom on eight nodes across the tooth base. Four different load cases were tested, each simulating a direction of stress that could have been experienced during prey capture: (i) tooth puncture with a single load force of 100 N directed at the tooth tip along the *z*-axis; (ii) mesial and distal load, representing lateral shake with a load force of 100 N spread across five nodes along the left lateral surface of the tooth crown; and (iii and iv) labial and lingual pull with a load force of 100 N spread across five nodes along the anterior and posterior surface of the tooth crown, respectively. These scenarios are largely similar to those in Whitenack *et al.* [46]. A standardized load force of 100 N was used for all simulations owing to the range of body sizes of the tested models. As this study seeks to compare only the relative performances of models, this standardization of loads without precise bite force data is appropriate to provide an accurate result [102].

Each mesh was subsequently imported into Abaqus (v. 6.10, Simulia) to be solved as a linear static model. The results were displayed as von Mises stress contour maps and as ridgeline plots showing the distribution of stress magnitudes for all elements.

### 3.5. Environmental context

To test for coevolutionary trends, we collected size data of contemporaneous ammonoids and chondrichthyans (electronic supplementary material, tables S2 and S3 and supplementary references). Measurements were partially taken from Klug *et al.* [103] and complemented by data of new material of both ammonoids and chondrichthyans. Furthermore, we assessed the change in sutural complexity of Palaeozoic ammonoids [104] using the number of sutural elements since complexity was possibly driven by predation pressure as demonstrated by Lemanis & Zlotnikov [105]. Both sutural complexity and the number of lobes of the ammonoid species with the most complex sutures of each stage were plotted (raw data are provided in the electronic supplementary material, tables S4 and S5 with the respective supplementary references). To test for a possible correlation between chondrichthyan body size and ammonoid conch size as well as sutural complexity, linear correlations (*r* and *p* uncorr) were performed using Past [106] (Excel sheet available on Zenodo: https://doi.org/10.5281/zenodo.14605615).

## 4. Results

**Figure 3.**
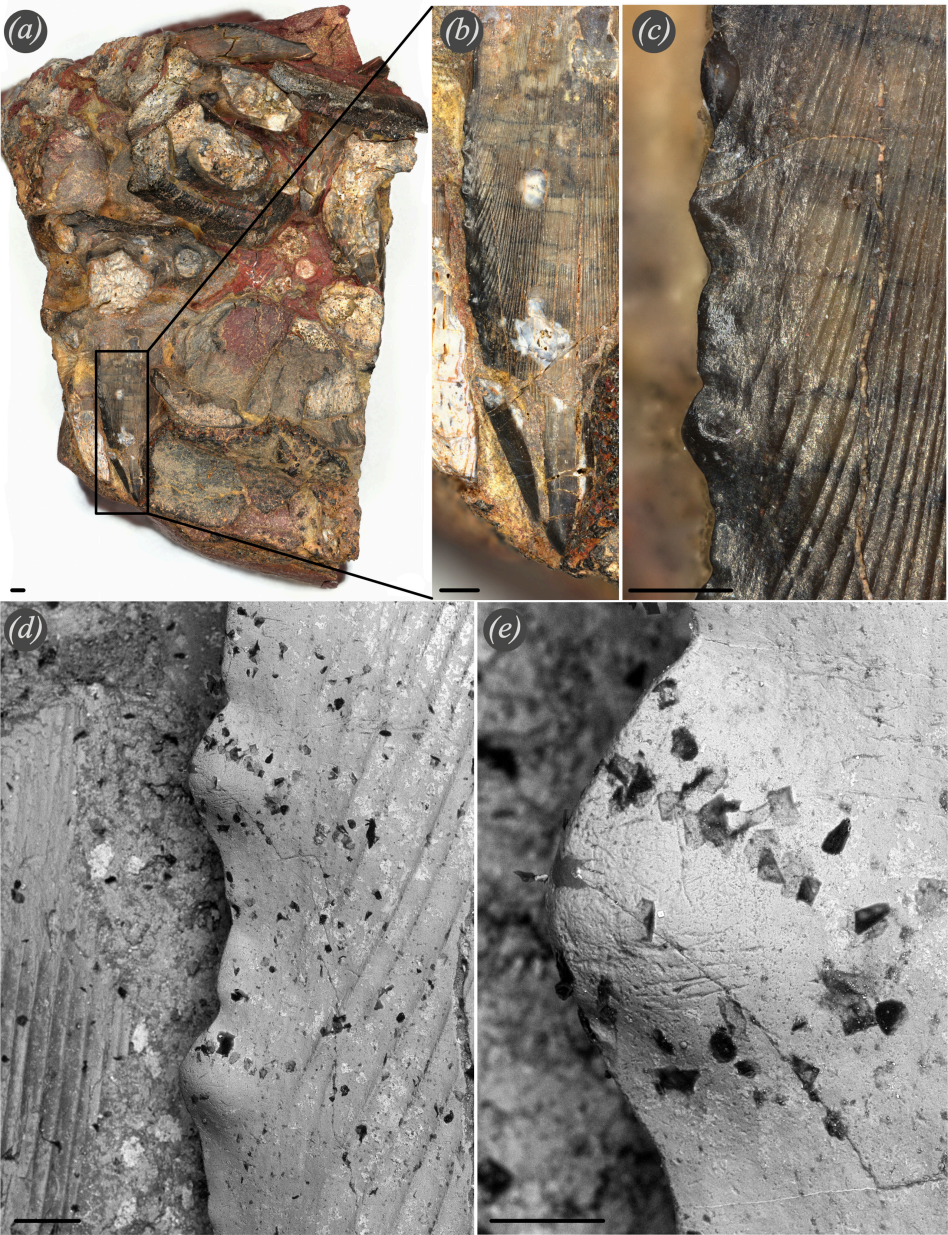
PIMUZ A/I 5263. *Ctenacanthus tumidus* from Madene El Mrakib, Famennian. (*a*) Overview of the entire block containing several *Ct. tumidus* teeth. The black box indicates which tooth is shown in detail in (*b*–*e*). Scale bar 1 mm. (*b*) Close-up of the apical part of the *Ct. tumidus* tooth showing the labial striation along the tooth as well as serration along the cutting edge, scale bar = 1 mm. (*c*) Close-up of the serrated cutting edge of *Ct. tumidus*, scale bar 0.5 mm. (*d*) SEM picture along the cutting edge of the tooth, scale bar 200 μm. (*e*) Detailed SEM picture of one tooth of the serrated cutting edge showing microwear pattern, scale bar 100 μm.

Wear facets and macroscopic scratches are visible on each tooth with the naked eye or a hand lens (figure 1; electronic supplementary material, figures S1 and S2). Counts of macroscopic scratches and their angles when oriented with the labial side facing dorsally and the apex away from the observer yield two main scratch directions. The apical parts of the wear facet often show somewhat vertical scratches while in the more abapical parts of the wear facets, the scratches are oriented rather horizontally (figure 1; electronic supplementary material, figure S3). Some teeth are worn down to about 30% of the original cusp height (electronic supplementary material, figure S1F,G) and show very strong and deep horizontal scratches. Comparing the teeth of the Late Famennian *Ct. concinnus* to *Ct. tumidus,* it is apparent that tooth wear is stronger in *Ct. concinnus*. By contrast, *Ct. tumidus* teeth show only slight degrees of wear along the serrated edges (figure 3), but no large wear facets are present.

### 4.1. Dental microwear texture analysis

**Figure 4.**
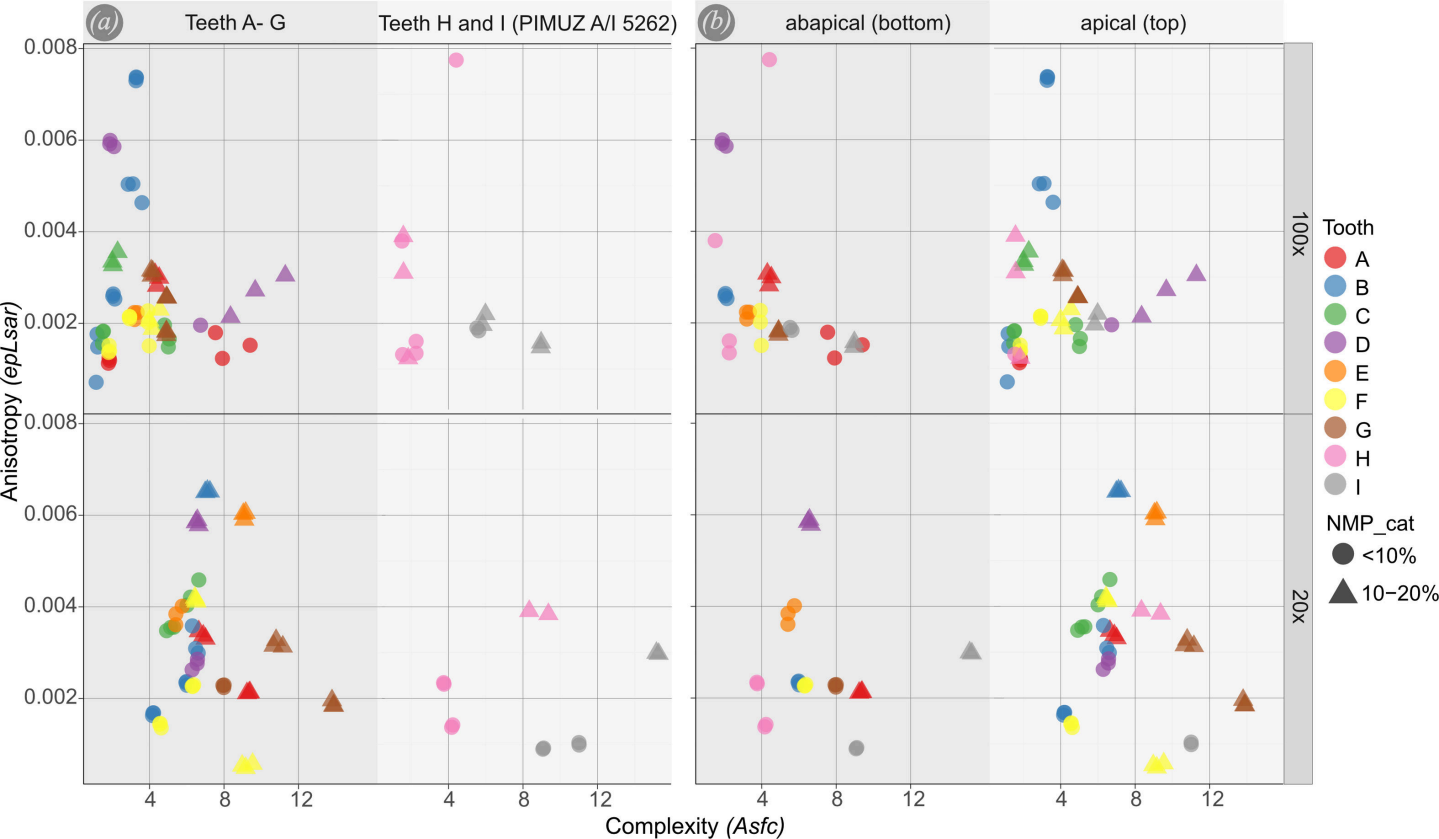
Results of the surface texture parameters *epLsar*—anisotropy and *Asfc*—complexity. The plots in (*a*) differentiate between the teeth of the two specimens. Teeth A–G, PIMUZ A/I 5253-5259 belong to one specimen and teeth H and I, PIMUZ A/I 5260 and 5261 belong to the skeleton PIMUZ A/I 5262. The plots in (*b*) differentiate between measurements that were taken in the apical parts of the wear facet and measurements that were taken in the abapical parts of the facet of each tooth. Top plots show data that were collected with a 100× objective. Bottom plots show data that were collected with a 20× objective. NMP_cat = non-measured points category; the percentage of unmeasured points of the measurement.

**Figure 5.**
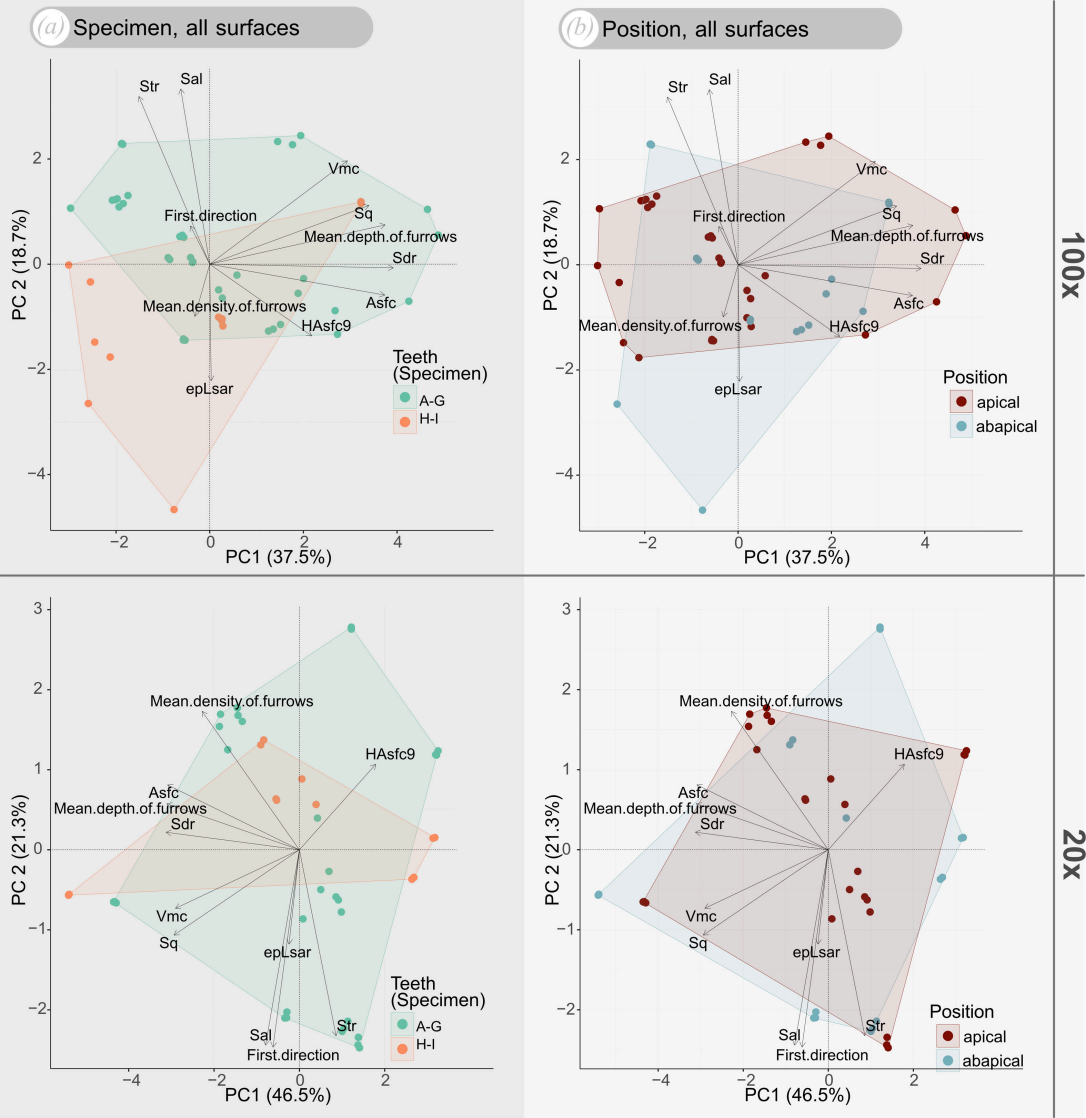
Principal components analysis (PCA) of the surface texture parameters displayed in the biplots, each for the first two principal components. (*a*) Two PCA plots differentiating between different specimens. Teeth A–G, PIMUZ A/I 5253-5259 belong to one specimen and teeth H and I, PIMUZ A/I 5260 and 5261, belong to the skeleton PIMUZ A/I 5262. (*b*) Two plots differentiating between the different positions (apical and abapical) that the measurements were taken on the wear facet of each tooth. The top plots represent measurements taken with a 100× objective. The bottom plots represent measurements taken with a 20× objective.

DMTA data did not show any clustering between the teeth or specimens (figure 4*a*). *Asfc* and *epLsar* values overall span wide ranges (figure 4). In many cases the measurements taken from one tooth plot closely together, but there are outliers (figure 4, e.g. teeth C and F). Regarding *Asfc*, values range from 0 to 16 while the variability and number of points decrease towards high values. The highest values at 20× are found in tooth G (PIMUZ A/I 5259), which appears very worn (electronic supplementary material, figure S1G). The highest values at 100× are found in tooth D (PIMUZ A/I 5256). However, there is no clear trend separating apical and abapical (top and bottom) measurements, or the two different specimens, neither at 100× nor at 20× objective magnifications. For *epLsar* (anisotropy), the values range from 0 to 0.008. Most measurements plot in the lower half of the plot (values of 0−0.002; figure 4). Regarding *epLsar* there is no pattern distinguishing measurements between the different specimens or between measurements taken in the apical and abapical parts of the wear facet. The values for different teeth in both specimens greatly overlap with few outliers (figure 4). PCA plots for all teeth and for the two different specimens show great degrees of overlap. For measurements taken with the 100× objective the overlap is less extensive (figure 5). Measurements taken from teeth A to G, as well as measurements taken in the apical part of the wear facet generally show higher values along PC2 and lower values along PC1 (figure 5).

### 4.2. Finite element analyses

**Figure 6.**
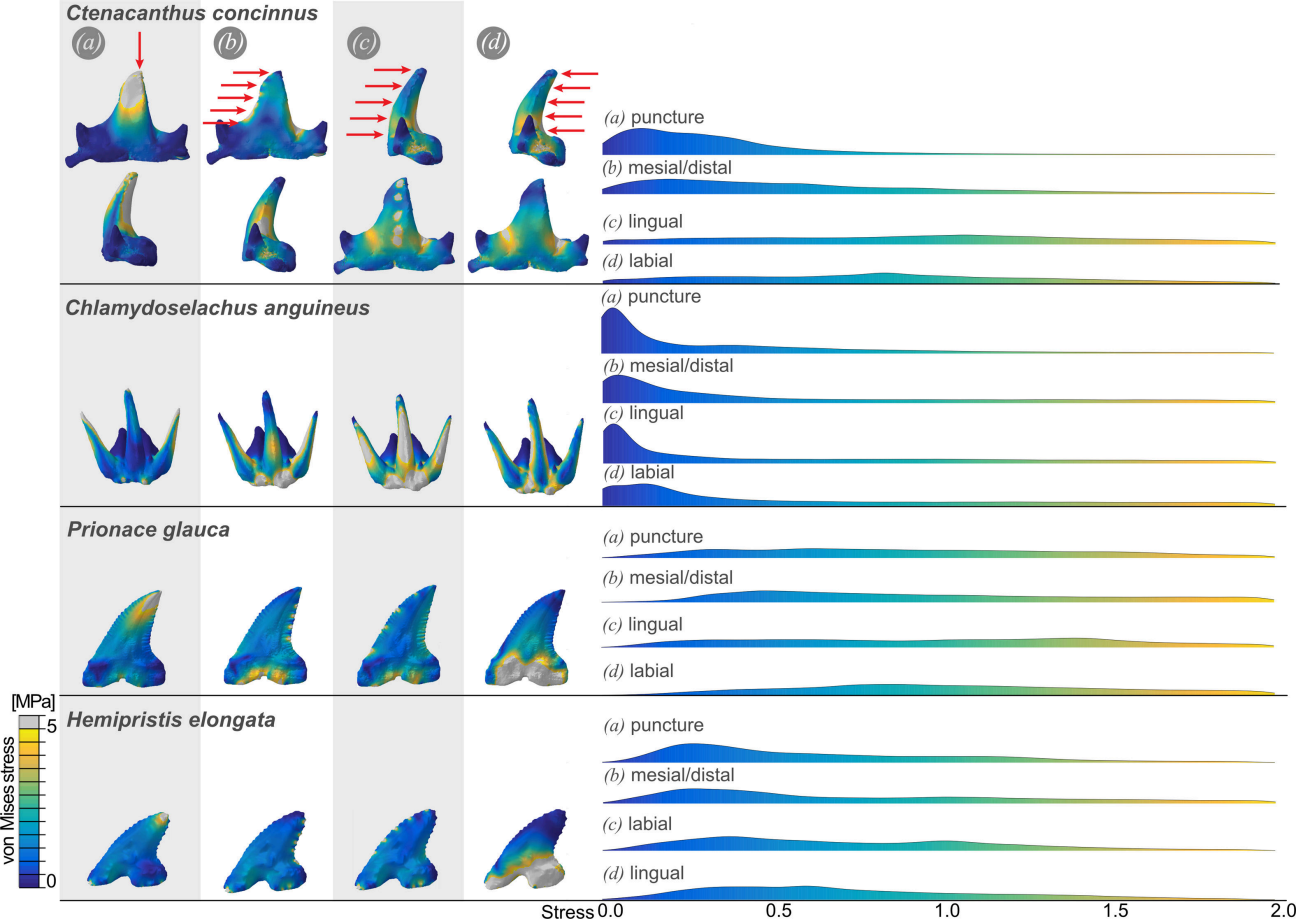
Finite element models of a *Ctenacanthus* tooth (PIMUZ A/I 5264, tooth J) and modern shark teeth, loaded from different directions. (*a*) Load applied to the tip, representing a puncture. (*b*) Load applied from distal and mesial, representing cutting through head shaking movements. (*c*) Load applied from labial and (*d*) lingual, representing holding of prey items.

Three different scenarios were simulated: load applied at the tip of the cusp, which represents puncture (figure 6*a*), load applied from the distal and mesial sides, which represents cutting and/or lateral head movements or headshaking (figure 6*b*), and load applied from the lingual and labial sides, which occurs when prey is grasped and held (figure 6*c,d*). The *Ct. concinnus* tooth reacts to a pointed load (figure 6a) with a concentration of stresses in the area where the load is applied. The stresses dissipate close to the loading site and do not extend to the whole tooth. When loads are applied from the distal and mesial sides, the stresses are highest where loads are applied but also dissipate quickly away from the loading sites, leaving the rest of the tooth relatively unstressed. In the third scenario, the stresses are distributed over the entire tooth, peaking in the gaps between the lateral cusps and the main cusp. This scenario recorded a larger number of elements with increased stresses, whereas the puncture and head-shaking scenarios recorded fewer elements with high stresses.

The modern shark teeth show different stress distribution patterns (figure 6). While *Ch. anguineus* shows high peaks in stress in almost all scenarios, stresses in *P. glauca* are generally more evenly distributed. The *H. elongata* stress distribution is most similar to *Ct. concinnus*. Under the puncture scenario, the stresses concentrate in one area. There is still some concentration in mesial/distal loads and a wider distribution in labial and lingual loads.

### 4.3. Environment

**Figure 7.**
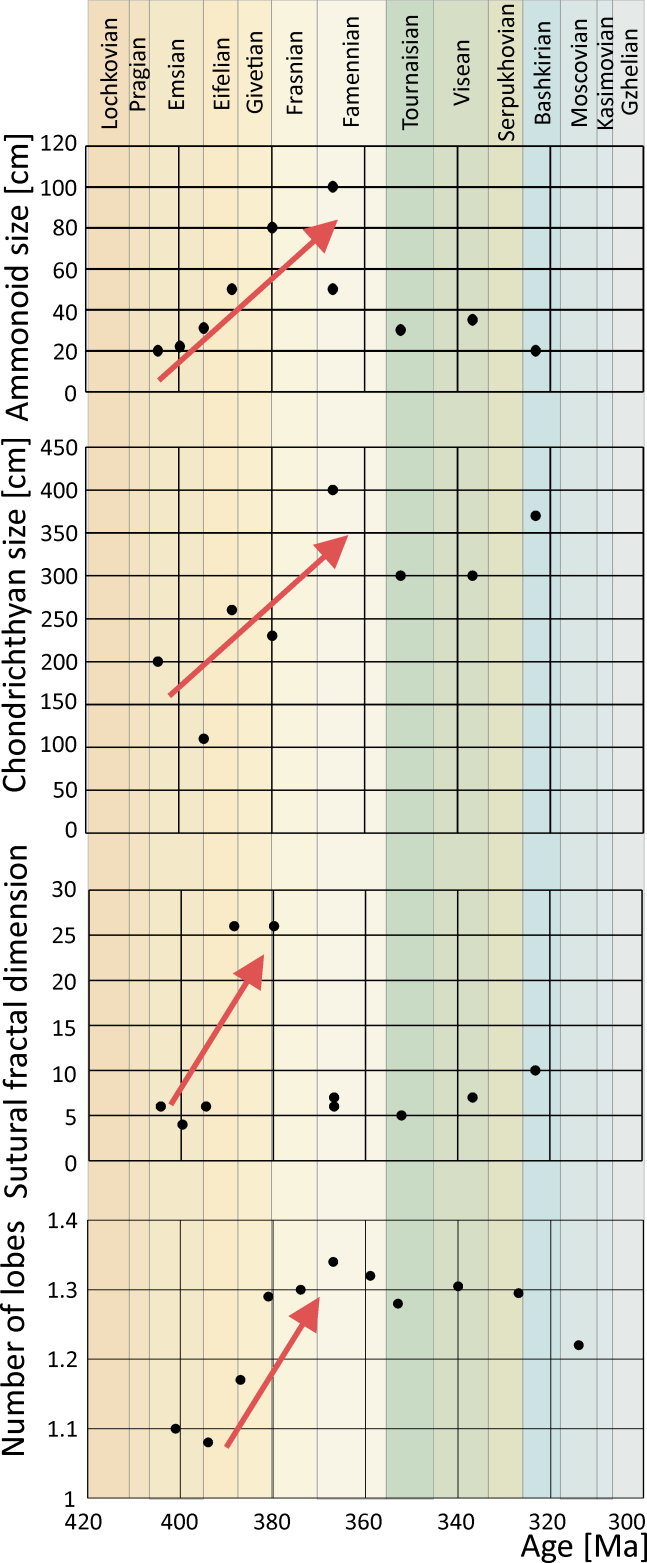
Chondrichthyan and ammonoid body size, and the numbers of lobes and sutural fractal dimension in ammonoid conchs, across the Devonian and Carboniferous, showing an increase in all four factors towards the Famennian. See also supplementary tables S1 to S4 and supplementary references. Excel sheet on Zenodo: https://doi.org/10.5281/zenodo.13772272.

Linear correlation of the values for ammonoid size, sutural dimension, number of lobes and the body size of chondrichthyans show an increase of all characters towards the Famennian and a sudden decrease at the Devonian/Carboniferous boundary (Kellwasser Event; figure 7). The best correlation for these values is present from the Emsian to Frasnian. Geologically older values show a weaker correlation (figure 7; Excel sheet Zenodo: https://doi.org/10.5281/zenodo.14605615).

## 5. Discussion

### 5.1. Tooth replacement rates and cutting edges

The teeth of chondrichthyans are their most important feature for capturing and processing prey and to keep long functionality, teeth are constantly replaced in modern sharks [41–43]. This polyphyodonty (constant replacement of teeth) is plesiomorphic within chondrichthyans [39,107,108]. Tooth replacement can be achieved in two different ways. Either the post-functional tooth is retained and moves beneath the skin on the outside of the head as exhibited by cladodonts [44], or the teeth are shed and replaced, as by neoselachians [41–43]. The term tooth replacement in the literature usually refers to the replacement of teeth by shedding. However, it is important to note that tooth replacement can also be achieved by retaining teeth that are not functional anymore [44]. Tooth retention is known from *Ctenacanthus* as well as *Cladoselache* and possibly the basal state that had to be overcome to establish fast tooth replacement in chondrichthyans [44].

The wide range of tooth sizes within the same jaw of *Ct. concinnus* in our material (from a width of less than 10 mm to over 50 mm) suggests that the teeth of these early chondrichthyans probably remained functional for a long time (arguably throughout the entire lifespan) [44] as the size of teeth from the same file is inversely proportional to the rate of replacement [38]. This is caused by the fact that during the growth of a specimen, the functional tooth size increases as well. Every newly formed tooth is larger than the preceding tooth in a file [38,42,44]. Assuming that one or potentially two or more teeth are functional at a time, tooth wear is only expected on these functional teeth but not on replacement teeth. Newberry [109] noticed extensive wear on cladodont teeth and interpreted these as having long functionality and slow replacement rates. Following on from Newberry [109] and William [44] on extensive tooth wear and tooth size, we propose *Ct. concinnus* had a slow tooth replacement. The absence of strong wear patterns and large wear facets in the teeth of *Ct. tumidus*, relative to *Ct. concinnus*, possibly relates to relatively higher tooth replacement rates. We cannot be certain of this since only few *Ct. tumidus* teeth from Morocco are available and we cannot determine whether these are functional teeth or possibly replacement teeth in hind rows, which are not worn. However, the main difference between the teeth of both species is the cutting edge (figures 2 and 3; for a description see the electronic supplementary material, S1). While the tooth edges of *Ct. concinnus* are sharp and without serrations, *Ct. tumidus* teeth possess a serrated cutting edge (figure 3; see [2, fig. 65]). The middle to late Famennian genus *Ctenacanthus* possibly represents the earliest instance of the evolution of serrated cutting edges in some species. A delicate serration probably wears down more quickly leading to a loss in functionality (at least partially). We hypothesize that the evolution of serrated cutting edges is correlated with the evolution from tooth retention as suggested for *Ctenacanthus* [44] to tooth replacement by shedding the functional teeth. This would allow the maintenance of a functional dentition as the functional tooth is shed and replaced quickly instead of being retained for long periods of time after the function is lost.

### 5.2. Tooth morphology and scratch patterns

The correlation between tooth morphology and feeding in chondrichthyans is still a matter of debate, as previous studies have produced conflicting results [26–29,32]. We therefore assess *Ct. concinnus* tooth morphology with reservation. All teeth possess a very similar morphology from small teeth to large ones and alongside the jaws (evident in other specimens as part of ongoing work). Therefore, we assume heterodonty [34] to be either absent or very subtle. We exclude durophagy as it is generally associated with the presence of tooth plates [4–6]. Following Cooper *et al.* [27] the overall robust tooth shape, the presence of several cusplets on the mesial and distal sides and the absence of serrations along the cutting edge most likely classifies *Ct. concinnus* as a clutching and grasping feeder [27]. Based on this study, the overall shape of the teeth of *Ct. concinnus* most likely points to a diet consisting of vertebrates. One characteristic feature of the teeth falling into this category in Cooper *et al.* [27] is the presence of serrations along the cutting edge which is absent in *Ct. concinnus*. Although the cutting edges are straight and sharp in rather unworn teeth of *Ct. concinnus*, a serrated edge is present in the contemporaneous *Ct. tumidus* (see above). Whitenack & Motta [26] tested the mechanics of shark teeth including some cladodont teeth of *Cladodus* which are morphologically similar to the teeth of *Ct. concinnus* and do not possess a serrated cutting edge. They demonstrated that *Cladodus* performed similarly well at puncturing and drawing as modern sharks and better than other fossil tooth morphotypes [26], but summarized that shark tooth morphology is not indicative of feeding mechanism. We do not exclude the option of cutting prey in *Ct. concinnus* although this difference might be a display of niche partitioning in Devonian ctenacanths. The study by Cooper *et al.* [27], as well as other studies regarding morphology and function, are mainly based on modern animals. For the reconstruction of feeding mechanisms in very early fossil stem group representatives such as *Ctenacanthus* these studies can only be used with reservation.

Another morphological tooth character of *Ct. concinnus* (as well as *Ct. tumidus*) is the presence of vertical parallel striated cristae on the lingual side which do not proceed in the apical part of the tooth (figure 2; electronic supplementary material, S1 and S2). In aquatic feeding mammals and reptiles there are similar ridges present, described as apicobasal enamel ridges [110]. While these ridges stabilize the teeth, their main function is to aid the efficiency of puncture, grip and removal during feeding [110]. In *Ct. concinnus* teeth, these ridges fade out towards the apex and are not visible in the wear facets (figures 1 and 2). Possibly, the ‘primitive’ mineralization of enameloid in early chondrichthyans favoured strong wear patterns and deep wear scratches as evident in the teeth of *Ct. concinnus* (figures 1 and 2; electronic supplementary material, figure S1). Enameloid in early chondrichthyans is known to be much simpler [111–114] than in neoselachians where the enameloid cap consists of three layers [36,113,115,116]. Despite strong wear, continued tooth use is possibly still occurring as can be seen in the recent example of the Port Jackson sharks where erosion of teeth is controlled by their mineralogy in a way that functionality is maintained [117]. Overall, the scratch orientations from *Ct. concinnus* teeth point towards puncture and shaking/cutting behaviours. Generally, the apical part of the tooth (figure 1*b*) shows vertical scratches, while the basal areas of the wear facet (figure 1*b*) show horizontal scratches. Some of the teeth are worn down until almost the base, while other teeth show extensive wear facets on the lingual sides (electronic supplementary material, figure S1). Different wear patterns might have been caused by the presence of several functional teeth per tooth file at a time. The positions of teeth within the tooth file cause different angles for each tooth, thus resulting in different tooth wear patterns and facet angles.

Based on morphology and scratch direction the shark either fed on two different kinds of prey or a spectrum of different prey animals. While smaller items could be procured and swallowed, larger ones would have been impossible to swallow whole. These items would have been secured by the upper teeth leaving vertical scratches, while side-to-side movement of the head facilitated the removal of large pieces of flesh resulting in horizontal scratches. The latter behaviour is known from recent marine apex predators such as the great white shark [118] or the tiger shark [13,118].

### 5.3. Biomechanical boundaries of the teeth—finite element analyses

The finite element models for the tooth of *Ct. concinnus* show the peak stresses at the tip of the tooth (where the initial contact with the prey occurs) in a puncture scenario (figure 6*a*). The rest of the tooth remains largely stress free. Hence, puncturing probably led to wear and breakage of the apex, which is apparent in *Ct. concinnus*. Puncture loads are expected when first biting into hard materials of prey items such as bone, cartilage, mineralized conchs, etc. Depending on the hardness of the item, damage is more or less likely. In a head-shaking scenario, the stresses also concentrate mainly where the load was applied (figure 6*b*). By contrast, when loads are applied from labial/lingual directions, the tooth shows higher stress values distributed throughout the entire tooth instead of isolated regions of peak stress (figure 6*c*,*d*). Strong labial and lingual loads are expected when a large prey animal (such as another shark) is grasped and held while it is withstanding the attack and trying to escape. Over a longer period, holding prey items would lead to larger amount of overall stress and therefore appear unlikely. When comparing the FEA results of *Ct. concinnus* to the stress patterns in the three modern sharks, the stress patterns seen in the tooth of the upper jaw of *H. elongata* appear most similar (figure 6). The feeding apparatus of *H. elongata* was studied in detail [119] and these authors stated that *H. elongata* most likely uses the upper dentition as a saw. Furthermore, they state that the processing of food items must be facilitated when the jaws are pronated, which leads to the upper teeth rotating outwards. From further fossils of *Ct. concinnus* (ongoing work), we know that the teeth are arranged around the jaw in such a way that the uppermost teeth most likely pointed outwards, thus making them similarly positioned and functional as in *H. elongata*. Taking these similarities into account, lateral and repeated movements of the head to process food items may have been a common feeding mechanism for ctenacanth chondrichthyans. Yet, further movements are essential to complete the feeding process. Accordingly, we suggest a combination of all tested scenarios, while lateral movements are crucial.

The *H. elongata* tooth is from the frontal upper jaw. In the case of the isolated *Ct. concinnus* tooth, we have no information on their original position. We are aware that the position possibly leads to different results. However, heterodonty is either low or absent in *Ct. concinnus* apart from size, meaning that stress distribution patterns of the FEA would not greatly differ.

### 5.4. Tooth wear analysis—dental microwear texture analysis

While the scratch directions and the FEA outputs indicate a puncture and shaking scenario, DMTA does not lead to such clear scenarios. The strong macrowear on the facets of *Ct. concinnus*, as well as the DMTA results, hint towards an opportunistic diet in *Ct. concinnus*. One exclusive, very specific movement of the jaw seems unlikely as it would result in a very specific DMTA pattern. By contrast, there seems to be no preferred overall microwear direction neither in general nor in the top or bottom parts of the wear facet and therefore no specific movement. Microwear seems to be non-discriminative which might be caused by the strong macrowear. When compared to other studies ([120, fig. 4]; [121, fig. 3]; [16, fig. 5]), the values for both *Asfc* and *epLsar* span a very wide range. In animals that only consume very hard or very soft food items, a much lower range in complexity would be expected. Strongly varying values for complexity, by contrast, suggest an opportunistic diet. Possibly, a large variety of prey items from animals with mineralized conchs or bones to softer-bodied animals generated the examined wear features. A wide range of anisotropy values suggests a combination of movements such as grasping, cutting and rotating as exhibited by some modern sharks [13,118]. Furthermore, a prey item itself possibly caused some scratches after being captured while trying to escape. Nevertheless, macroscopic scratches generally show two major orientations as mentioned earlier (vertical scratches in the apical, horizontal scratches in the abapical part of the wear facet; figure 2).

Unfortunately, we do not have data to compare the DMTA results to other groups of early vertebrates or chondrichthyans in a standardized fashion. DMTA was here applied for the first time, to our knowledge, to Palaeozoic vertebrates and there is no published data of comparably old vertebrates. The only existing data for chondrichthyans are from McLennan & Purnell [31] and Weber *et al.* [16]. However, these data were acquired using different microscopes (Alicona Infinite Focus G4b and Nanofocus µsurf Custom, respectively). Depending on the type of microscope, the output differs and therefore cannot be compared directly. Arman *et al.* [122] developed a template to deal the overcome problem. However, the results from these instruments are too different to be homogenized using this template. Therefore, quantitative comparisons of the data are not possible. Nevertheless, a qualitative comparison is informative. Weber *et al.* [16] used a sample set comprising teeth from 24 recent and 12 fossil (Oligocene to Pleistocene) neoselachian species. They furthermore tested taphonomic alterations on the enameloid of shark teeth in an experimental setting. They concluded that there is no obvious relationship between ante-mortem tooth wear in the recent shark teeth and their diet. Furthermore, tooth wear patterns in the fossil species appear to be alternatively caused by transport, or possibly by a very different kind of diet including harder items [16]. McLennan & Purnell [31] analysed data of the sandtiger shark, *Carcharias taurus*. By using DMTA on teeth of wild and aquarium-fed *Ca. taurus*, this study showed that DMTA can be a useful tool for tooth wear analysis. However, they point out that analyses that are based on single isolated teeth, rather than those from jaws, can potentially detect differences between populations and species with different diets but with a low sensitivity [31]. Both these studies were applied to neoselachians teeth. Comparing DMT data from extant neoselachians and Devonian chondrichthyans does not make sense because of the differences in tooth replacement rates and in environmental contexts. To distinguish between different kinds of diet, more tooth wear data of Devonian vertebrates would be needed, which unfortunately is problematic owing to the lack of well-preserved fossils of that time showing tooth wear.

Importantly, it must be kept in mind that the position of each fossil tooth in the jaw is unknown. Tooth positions change while they move out of the dental lamina to the crest of the jaw and then to the jaw margin [38,42,44]. The pattern of anisotropy probably also changed with tooth position in the jaw, as also noticed by Weber *et al.* [16], since the teeth are used and worn differently depending on their position. For example, great white sharks use mainly their anterior teeth to capture prey [118], while the posterior teeth are rarely involved. Also, lower jaw orientation might change during biting, as demonstrated by Frey *et al.* [63] for the symmoriiform *Ferromirum*. Furthermore, Weber *et al.* [16] mention ontogenetic variations in DMT but were not able to specify what causes these variations. Possible variables leading to differences in DMT can be changes to tooth replacement rate, bite force, diet and/or habitat with age. Ontogenetic changes in such variables, as well as tooth position on the jaw, are unknown in *Ct. concinnus*.

In conclusion, DMTA data from these early chondrichthyans do not show a distinctive pattern but suggest an opportunistic diet. The data presented here can serve as comparisons for future studies that deal with DMTA in early vertebrates.

### 5.5. Environmental context

**Figure 8.**
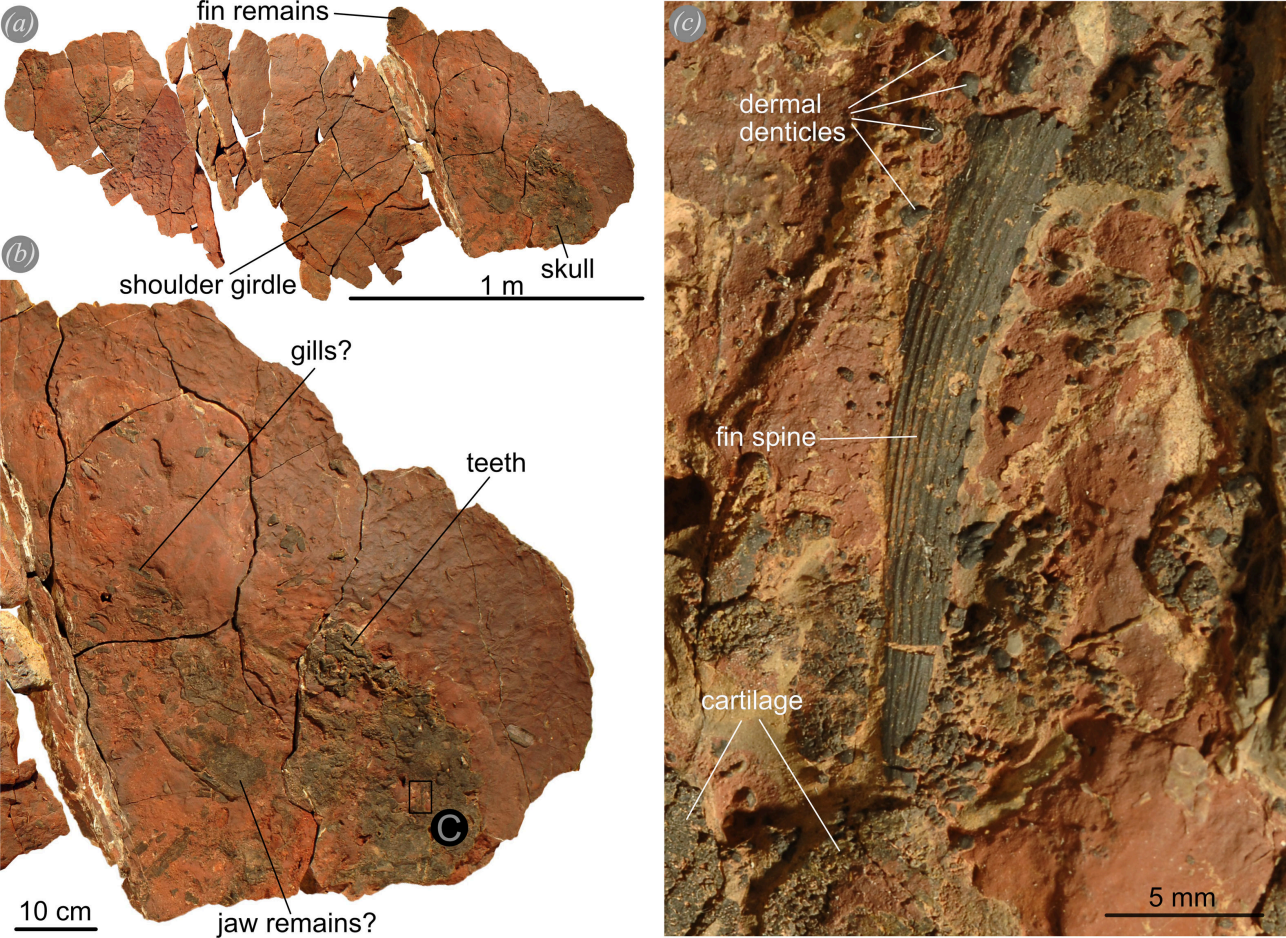
PIMUZ A/I 5338. Ctenacanth fossil from the Famennian of the Anti-Atlas, Morocco, with a preserved fin spine of another chondrichthyan in its mouth. (*a*) Overview of the entire fossil. (*b*) Overview of the skull, the black box indicates where (*c*), a chondrichthyan fin spine of a different taxa, is located.

The Devonian strata of Morocco bear abundant macro and micro remains of vertebrates including chondrichthyans [22,62,63], placoderms [60,61,64,65,123], acanthodians [124] and actinopterygians ([22,125], electronic supplementary material, figure S14). The highly fossiliferous *Gonioclymenia* layer yields many vertebrate and invertebrate species, including jawed fishes [51,52,126–128], ammonoids [129–132] and conodonts [129,133]. The extremely abundant index genus *Gonioclymenia* reached conch diameters of over half a metre and *Costaclymenia* even up to 1 m [134] in the Anti-Atlas. Given the abundance of a variety of vertebrates and invertebrates, *Ctenacanthus* possibly fed on an array of hard-shelled or bony organisms, which is supported by DMTA. One unpublished ctenacanth fossil (PIMUZ A/I 5338) preserves a fin spine of another chondrichthyan in its mouth (figure 8) suggesting that smaller chondrichthyans were part of the *Ctenacanthus* diet. Another potential food source might have been smaller placoderms, such as *Amazichthys* [65] or juvenile individuals of other arthrodires, although they are not very abundant in the *Gonioclymenia* layer. Furthermore, there is direct evidence that actinopterygians have been part of the diet of another chondrichthyan, *Maghriboselache,* from the Late Devonian of Morocco, as shown in Klug *et al*. [19] (electronic supplementary material, figure S14). Besides these, the high abundance and increased sutural complexity (frilling of septa) of clymeniid ammonoids in the same layers suggests that ctenacanths preyed on these omnipresent ammonoids even though no direct evidence is available. This hypothesis is supported by the fact that during the Devonian, both chondrichthyan body size [135] and ammonoid conch size [103] increased (figure 7). Furthermore, the number of lobes and the fractal complexity increased until the Late Devonian [104,136]. The sutural complexity was long believed to help regulate buoyancy and increase resistance towards hydrostatic pressure. However, Lemanis & Zlotnikov [105] interpreted the higher sutural complexity as a result of a positive selection for conchs to be more resistant against point loads. Hypothetically, this increase in size and sutural complexity reflects co-evolution between increased predation pressure from ctenacanths and other gnathostomes, resulting in selection for larger body sizes, which in turn allowed them to prey on these large animals (Red Queen hypothesis [137]). Similar co-evolution was observed by Sallan *et al.* [135] which investigated the complexity of calyxes in crinoids in the early Carboniferous and the transition to more durophagous-like dentitions in chondrichthyans. Finally, a regurgitate containing numerous small ammonoid shell shards from the latest Famennian of the southern Maїder [138] with a small ctenacanth occurring in the same layer [139] supports the hypothesis that sharks may have fed on ammonoids. However, a preliminary statistical analysis showed only a weak correlation between body size increases of ammonoids and chondrichthyans, or the increase in sutural complexity and chondrichthyan body size (electronic supplementary material, tables S3–S5; Excel sheet available on Zenodo: https://doi.org/10.5281/zenodo.14605615).

## 6. Conclusions

Combining approaches to reconstruct the diet of the Devonian chondrichthyan *Ct. concinnus* led to a series of results:

- scratches indicate grasping, puncturing and lateral head movements (to cut prey);
- tooth morphology indicates clutching or grasping feeding;
- FEA supports lateral head movements in combination with puncturing and holding;
- DMTA suggests an opportunistic diet and a variety of head movements;
- a parallel increase in chondrichthyan body size and ammonoid body size and sutural complexity hints towards a Red Queen effect; and
- placoderms, chondrichthyans, actinopterygians or conodonts are further possible prey.

Taken together, *Ctenacanthus* most likely used an array of feeding mechanisms and was opportunist, catching the easiest (least dangerous) and most common prey. Prey items were probably captured and processed using an array of different movements, such as puncturing and cutting by horizontal or rotational head movements. Generally, the correlation between tooth morphology and function remains a matter of discussion. However, serrated cutting edges are usually correlated with cutting pieces off larger items. Serrations along tooth edges are present in *Ct. tumidus* but absent in *Ct. concinnus*. We thus include cutting as a possible feeding mechanism for the genus. This raises the question of niche partitioning, not only among Late Devonian fishes and Famennian chondrichthyans, but also within the genus. Of all ctenacanth specimens available to us, *Ct. concinnus* are by far the largest. This size difference is counterintuitive as *Ct. tumidus* serrated edges would have facilitated predation of larger prey items by reducing their size. However, this could be linked to the greater abundance of *Ct. concinnus* remains in the Devonian Anti-Atlas. Further material and research are needed to improve our understanding of trophic levels and niche partitioning of Late Devonian fishes and particularly chondrichthyans.

Combining DMTA, FEA and morphological and environmental data showcased the difficulties in robustly investigating the functional morphology and dietary ecology of early Palaeozoic vertebrates such as Devonian chondrichthyans. Many unknown factors can bias the results of each approach. This shows that relying solely on one method could produce wrong assumptions and the importance of combining different methodologies in palaeontological studies.

Regarding feeding mechanisms and diet, we cannot make definitive statements with the information available to us. However, it appears that ctenacanthids used all available food sources that provided enough nutrients such as smaller sharks, large ammonoids and speculatively also small (juvenile) placoderms, making *Ctenacanthus* an apex predator of the Devonian.

## Data Availability

There are two repositories on Zenodo: data used for DMTA, FEA and further: [140] and GitHub repository: [141] Supplementary material is available online [142].

## References

[1] Cooper JA, Pimiento C. 2024 The rise and fall of shark functional diversity over the last 66 million years. Glob. Ecol. Biogeogr. **33**, e13881. (10.1111/geb.13881)

[2] Ginter M, Hampe O, Duffin CJ. 2010 Chondrichthyes Paleozoic Elasmobranchii: Teeth: Handbook of Paleoichthyology. vol. 3D. München, Germany: Verlag Dr. Friedrich Pfeil.

[3] Smith MM, Johanson Z, Underwood C, Diekwisch TGH. 2013 Pattern formation in development of chondrichthyan dentitions: a review of an evolutionary model. Hist. Biol. **25**, 127–142. (10.1080/08912963.2012.662228)

[4] Wilga CD, Motta PJ. 2000 Durophagy in sharks: feeding mechanics of the hammerhead Sphyrna Tiburo. J. Exp. Biol. **203**, 2781–2796. (10.1242/jeb.203.18.2781) 10952878

[5] Summers AP. 2000 Stiffening the stingray skeleton – an investigation of durophagy in Myliobatid stingrays (Chondrichthyes, Batoidea, Myliobatidae). J. Morphol. **243**, 113–126. (10.1002/(SICI)1097-4687(200002)243:23.0.CO;2-A) 10658196

[6] Shimada K. 2012 Dentition of Late Cretaceous shark, Ptychodus mortoni (Elasmobranchii, Ptychodontidae). J. Vertebr. Paleontol. **32**, 1271–1284. (10.1080/02724634.2012.707997)

[7] Motta PJ, Wilga CD. 2001 Advances in the study of feeding behaviors, mechanisms, and mechanics of sharks. In Developments in environmental biology of fishes the behavior and sensory biology of elasmobranch fishes: an anthology in memory of Donald Richard Nelson (eds T Tricas, S Gruber), pp. 131–156. Dordrecht, The Netherlands: Springer Netherlands. (10.1007/978-94-017-3245-1_10)

[8] Motta PJ. 2004 Prey capture behavior and feeding mechanics of elasmobranchs. In Biology of sharks and their relatives (eds JC Carrier, JA Musick, MR Heithaus), pp. 165–202. Boca Raton: CRC Press. (10.1201/9780203491317.ch6)

[9] Wetherbee B, Gruber S, Cortés E. 1990 Diet, feeding habits and consumption in sharks, with special reference to the lemon shark, Negaprion brevirostris. Natl Oceanogr. Atmos. Adm. Tech. Rep. NMFS **1990**, 29–47.

[10] Lowe CG, Wetherbee BM, Crow GL, Tester AL. 1996 Ontogenetic dietary shifts and feeding behavior of the tiger shark, Galeocerdo cuvier, in Hawaiian waters. Environ. Biol. Fishes **47**, 203–211. (10.1007/bf00005044)

[11] Curtis T *et al*. 2006 Observations on the behavior of white sharks scavenging from a whale carcass at Point Reyes, California. Calif. Fish Game **92**, 113–124.

[12] Markaida U, Sosa-Nishizaki O. 2010 Food and feeding habits of the blue shark Prionace glauca caught off Ensenada, Baja California, Mexico, with a review on its feeding. J. Mar. Biol. Assoc. UK **90**, 977–994. (10.1017/s0025315409991597)

[13] Clua E, Chauvet C, Read T, Werry JM, Lee SY. 2013 Behavioural patterns of a tiger shark (Galeocerdo cuvier) feeding aggregation at a blue whale carcass in Prony Bay, New Caledonia. Mar. Freshw. Behav. Physiol. **46**, 1–20. (10.1080/10236244.2013.773127)

[14] McNeil B, Lowry D, Larson S, Griffing D. 2016 Feeding behavior of subadult sixgill sharks (Hexanchus griseus) at a bait station. PLoS ONE **11**, e0156730. (10.1371/journal.pone.0156730) 27243237 PMC4887027

[15] Moyer JK, Shannon SF, Irschick DJ. 2019 Bite performance and feeding behaviour of the sand tiger shark Carcharias taurus. J. Fish Biol. **95**, 881–892. (10.1111/jfb.14086) 31265127

[16] Weber K, Winkler DE, Kaiser TM, Žigaitė Ž, Tütken T. 2021 Dental microwear texture analysis on extant and extinct sharks: ante- or post-mortem tooth wear? Palaeogeogr. Palaeoclimatol. Palaeoecol. **562**, 110147. (10.1016/j.palaeo.2020.110147)

[17] Hunt AP, Lucas SG, Milan J, Spielmann JA. 2012 Vertebrate coprolite studies: a status as prospectus. New Mexico Museum of Natural History and Science, Bulletin **57**, 1–24.

[18] Hunt AP, Lucas SG. 2012 Classification of vertebrate coprolites and related trace fossils. New Mexico Museum of Natural History and Science, Bulletin **57**, 137–146.

[19] Kriwet J, Witzmann F, Klug S, Heidtke UHJ. 2008 First direct evidence of a vertebrate three-level trophic chain in the fossil record. Proc. R. Soc. B **275**, 181–186. (10.1098/rspb.2007.1170) PMC2596183 17971323

[20] Chevrinais M, Jacquet C, Cloutier R. 2017 Early establishment of vertebrate trophic interactions: food web structure in Middle to Late Devonian fish assemblages with exceptional fossilization. Bull. Geosci. **92**, 491–510. (10.3140/bull.geosci.1651)

[21] Klug C, Schweigert G, Hoffmann R, Weis R, De Baets K. 2021 Fossilized leftover falls as sources of palaeoecological data: a ‘pabulite’ comprising a crustacean, a belemnite and a vertebrate from the Early Jurassic Posidonia Shale. Swiss J. Palaeontol. **140**, 10. (10.1186/s13358-021-00225-z) 34721282 PMC8549986

[22] Klug C, Coates M, Frey L, Greif M, Jobbins M, Pohle A, Lagnaoui A, Haouz WB, Ginter M. 2023 Broad snouted cladoselachian with sensory specialization at the base of modern chondrichthyans. Swiss J. Palaeontol. **142**, 2. (10.1186/s13358-023-00266-6) 37009301 PMC10050047

[23] Zatoń M, Rakociński M. 2014 Coprolite evidence for carnivorous predation in a Late Devonian pelagic environment of southern Laurussia. Palaeogeogr. Palaeoclimatol. Palaeoecol. **394**, 1–11. (10.1016/j.palaeo.2013.11.019)

[24] Noriega JI, Cione AL, Aceñolaza FG. 2007 Shark tooth marks on Miocene balaenopterid cetacean bones from Argentina. Neues Jahrb. Für Geol. Und Paläontol. Abh. **245**, 185–192. (10.1127/0077-7749/2007/0245-0185)

[25] Pobiner B. 2008 Paleoecological information in predator tooth marks. J. Taphon. **6**, 373–397. (10.3390/info6010001)

[26] Whitenack LB, Motta PJ. 2010 Performance of shark teeth during puncture and draw: implications for the mechanics of cutting. Biol. J. Linn. Soc. **100**, 271–286. (10.1111/j.1095-8312.2010.01421.x)

[27] Cooper JA, Griffin JN, Kindlimann R, Pimiento C. 2023 Are shark teeth proxies for functional traits? A framework to infer ecology from the fossil record. J. Fish Biol. **103**, 798–814. (10.1111/jfb.15326) 36651356

[28] Bazzi M, Campione NE, Ahlberg PE, Blom H, Kear BP. 2021 Tooth morphology elucidates shark evolution across the end-Cretaceous mass extinction. PLoS Biol. **19**, e3001108. (10.1371/journal.pbio.3001108) 34375335 PMC8354442

[29] Paredes-Aliaga MV, Botella H, Romero A. 2024 Testing dental microwear as a proxy for characterising trophic ecology in fossil elasmobranchs (Chondrichthyans). Swiss J. Palaeontol. **143**, 29. (10.1186/s13358-024-00322-9)

[30] Paredes-Aliaga MV, Herraiz JL. 2024 Analysing trophic competition in †Otodus megalodon and Carcharodon carcharias through 2D-SEM dental microwear. Span. J. Palaeontol. **39**, 102. (10.7203/sjp.28830)

[31] McLennan LJ, Purnell MA. 2021 Dental microwear texture analysis as a tool for dietary discrimination in elasmobranchs. Sci. Rep. **11**, 2444. (10.1038/s41598-021-81258-9) 33510241 PMC7844039

[32] Ciampaglio CN, Wray GA, Corliss BH. 2005 A toothy tale of evolution: convergence in tooth morphology among marine Mesozoic–Cenozoic sharks, reptiles, and mammals. Sediment. Rec. **3**, 4–8. (10.2110/sedred.2005.4.4)

[33] Jacquemin SJ. 2016 Quantifying heterodonty in the late Devonian (Upper Famennian) sharks Cladoselache and Ctenacanthus from the Ohio Shale, USA. PalArchs J. Vertebr. Palaeontol. **13**, 1–20.

[34] Türtscher J, Jambura PL, López‐Romero FA, Kindlimann R, Sato K, Tomita T, Kriwet J. 2022 Heterodonty and ontogenetic shift dynamics in the dentition of the tiger shark Galeocerdo cuvier (Chondrichthyes, Galeocerdidae). J. Anat. **241**, 372–392. (10.1111/joa.13668) 35428996 PMC9296035

[35] Schulz E, Calandra I, Kaiser TM, Winkler DE. 2020 A brief history of quantitative wear analyses with an appeal for a holistic view on dental wear processes. In Mammalian teeth – form and function (eds T Martin, W Koenigswald), pp. 44–53. Verlag Dr. Friedrich Pfeil, DE.

[36] Enax J, Prymak O, Raabe D, Epple M. 2012 Structure, composition, and mechanical properties of shark teeth. J. Struct. Biol. **178**, 290–299. (10.1016/j.jsb.2012.03.012) 22503701

[37] Loch C, Simões-Lopes PC. 2013 Dental wear in dolphins (Cetacea: Delphinidae) from southern Brazil. Arch. Oral Biol. **58**, 134–141. (10.1016/j.archoralbio.2012.08.002) 22939372

[38] Botella H, Valenzuela‐Ríos JI, Martínez‐Pérez C. 2009 Tooth replacement rates in early chondrichthyans: a qualitative approach. Lethaia **42**, 365–376. (10.1111/j.1502-3931.2009.00152.x)

[39] Tucker AS, Fraser GJ. 2014 Evolution and developmental diversity of tooth regeneration. Semin. Cell Dev. Biol. **25–26**, 71–80. (10.1016/j.semcdb.2013.12.013) 24406627

[40] Schnetz L, Pfaff C, Kriwet J. 2016 Tooth development and histology patterns in lamniform sharks (Elasmobranchii, Lamniformes) revisited. J. Morphol. **277**, 1584–1598. (10.1002/jmor.20597) 27587092

[41] Moss SA. 1972 Tooth replacement and body growth rates in the smooth dogfish, Mustelus canis (Mitchill). Copeia **1972**, 808–811. (10.2307/1442738)

[42] Luer CA, Blum PC, Gilbert PW. 1990 Rate of tooth replacement in the nurse shark, Ginglymostoma cirratum. Copeia **1990**, 182–191. (10.2307/1445834)

[43] Correia JP. 1999 Tooth loss rate from two captive sandtiger sharks (Carcharias taurus). Zoo Biol. **18**, 313–317. (10.1002/(sici)1098-2361(1999)18:43.0.co;2-d)

[44] Williams ME. 2001 Tooth retention in cladodont sharks: with a comparison between primitive grasping and swallowing, and modern cutting and gouging feeding mechanisms. J. Vertebr. Paleontol. **21**, 214–226. (10.1671/0272-4634(2001)021[0214:tricsw]2.0.co;2)

[45] Reif WE. 1982 Evolution of dermal skeleton and dentition in vertebrates. In Evolutionary biology (ed. MK Hecht), pp. 287–368. New York: Plenum press. (10.1007/978-1-4615-6968-8_7)

[46] Whitenack LB, Simkins DC, Motta PJ. 2011 Biology meets engineering: the structural mechanics of fossil and extant shark teeth. J. Morphol. **272**, 169–179. (10.1002/jmor.10903) 21210488

[47] Agassiz L. 1837 De genre Ctenacanthus Agass’. Contenant l’Histoire de l’Ordre de Placoïdes. Rech. Sur. Poissons Foss. **3**, 10–12.

[48] Hodnett JPM, Elliott DK, Olson TJ, Wittke JH. 2012 Ctenacanthiform sharks from the Permian Kaibab Formation, northern Arizona. Hist. Biol. **24**, 381–395. (10.1080/08912963.2012.683193)

[49] Maisey JG, Bronson AW, Williams RR, McKinzie M. 2017 A Pennsylvanian ‘supershark’ from Texas. J. Vertebr. Paleontol. **37**, e1325369. (10.1080/02724634.2017.1325369)

[50] Patterson C, White EI. 1965 The phylogeny of the chimaeroids. Phil. Trans. R. Soc. Lond. B **249**, 101–219. (10.1098/rstb.1965.0010)

[51] Ginter M, Hairapetian V, Klug C. 2002 Famennian chondrichthyans from the shelves of North Gondwana. Acta Geol. Pol. **52**, 169–215.

[52] Derycke C. 2017 Paléobiodiversité des gnathostomes (chondrichthyens, acanthodiens et actinoptérygiens) du Dévonien du Maroc (NW Gondwana). In Paléontologie des vertébrés du maroc: état des connaissances (ed. S Zouhri), pp. 47–78, vol. 180. Paris: Mémoires de la Société Géologique de France.

[53] Carr RK, Jackson GL. 2008 The vertebrate fauna of the Cleveland shale member (Famennian) of the Ohio shale. Ohio Geological Survey Guidebook 22, Guide to the Geology and Paleontologyof the Cleveland Member of the Ohio Shale (in press). Cleveland, Ohio, October 15-18: Prepared for the 68th Annual Meetingof the Society of Vertebrate Paleontology.

[54] Torices A, Wilkinson R, Arbour VM, Ruiz-Omeñaca JI, Currie PJ. 2018 Puncture-and-pull biomechanics in the teeth of predatory coelurosaurian dinosaurs. Curr. Biol. **28**, 1467–1474.(10.1016/j.cub.2018.03.042) 29706515

[55] Becker RT, Marshall JEA, Da Silva AC, Agterberg FP. 2020 The Devonian Period. In Geologic time scale 2020 (eds FM Gradstein, JG Ogg, MD Schmitz, GM Ogg), pp. 733–810. Amsterdam, The Netherlands: Elsevier. (10.1016/B978-0-12-824360-2.00022-X)

[56] Wendt J, Aigner T. 1985 Facies patterns and depositional environments of Palaeozoic cephalopod limestones. Sediment. Geol. **44**, 263–300. (10.1016/0037-0738(85)90016-8)

[57] Wendt J. 2021 Middle and Late Devonian paleogeography of the eastern Anti-Atlas (Morocco). Int. J. Earth Sci. **110**, 1531–1544. (10.1007/s00531-021-02028-6)

[58] Lehman JP. 1956 Les Arthrodires du Dévonien supérieur du Tafilalt (Sud Marocain). Notes. et. Mém. du. Serv. Géol. du. Maroc. **129**, 1–70.

[59] Lehman JP. 1976 Nouveaux poissons fossiles du Dévonien du Maroc. Ann. Paléohistol. vertébrés. **62**, 1–34. (10.1016/j.annpal.2006.03.009)

[60] Rücklin M. 2011 First selenosteid placoderms from the eastern Anti‐Atlas of Morocco; osteology, phylogeny and palaeogeographical implications. Palaeontology **54**, 25–62. (10.1111/j.1475-4983.2010.01026.x)

[61] Rücklin M, Long JA, Trinajstic K. 2015 A new selenosteid arthrodire (‘Placodermi’) from the Late Devonian of Morocco. J. Vertebr. Paleontol. **35**, e908896. (10.1080/02724634.2014.908896)

[62] Frey L, Coates M, Ginter M, Hairapetian V, Rücklin M, Jerjen I, Klug C. 2019 The early elasmobranch Phoebodus: phylogenetic relationships, ecomorphology and a new time-scale for shark evolution. Proc. R. Soc. B **286**, 20191336. (10.1098/rspb.2019.1336) PMC6790773 31575362

[63] Frey L, Coates MI, Tietjen K, Rücklin M, Klug C. 2020 A symmoriiform from the Late Devonian of Morocco demonstrates a derived jaw function in ancient chondrichthyans. Commun. Biol. **3**, 681. (10.1038/s42003-020-01394-2) 33203942 PMC7672094

[64] Jobbins M, Rücklin M, Argyriou T, Klug C. 2021 A large Middle Devonian eubrachythoracid ‘placoderm’ (Arthrodira) jaw from northern Gondwana. Swiss J. Palaeontol. **140**, 2. (10.1186/s13358-020-00212-w) 33488510 PMC7809001

[65] Jobbins M, Rücklin M, Ferrón HG, Klug C. 2022 A new selenosteid placoderm from the Late Devonian of the eastern Anti-Atlas (Morocco) with preserved body outline and its ecomorphology. Front. Ecol. Evol. **10**. (10.3389/fevo.2022.969158)

[66] Calandra I, Labonne G, Schulz-Kornas E, Kaiser TM, Montuire S. 2016 Tooth wear as a means to quantify intra-specific variations in diet and chewing movements. Sci. Rep. **6**, 34037. (10.1038/srep34037) 27658531 PMC5034321

[67] Calandra I, Merceron G. 2016 Dental microwear texture analysis in mammalian ecology. Mammal Rev. **46**, 215–228. (10.1111/mam.12063)

[68] Damuth J, Janis CM. 2014 A comparison of observed molar wear rates in extant herbivorous mammals. Ann. Zool. Fenn. **51**, 188–200. (10.5735/086.051.0219)

[69] DeSantis LRG. 2016 Dental microwear textures: reconstructing diets of fossil mammals. Surf. Topogr. **4**, 023002. (10.1088/2051-672X/4/2/023002)

[70] Kaiser TM, Clauss M, Schulz-Kornas E. 2015 A set of hypotheses on tribology of mammalian herbivore teeth. Surf. Topogr. **4**, 014003. (10.1088/2051-672X/4/1/014003)

[71] Mihlbachler MC, Rivals F, Solounias N, Semprebon GM. 2011 Dietary change and evolution of horses in North America. Science **331**, 1178–1181. (10.1126/science.1196166) 21385712

[72] Rivals F, Uno KT, Bibi F, Pante MC, Njau J, de la Torre I. 2018 Dietary traits of the ungulates from the HWK EE site at Olduvai Gorge (Tanzania): diachronic changes and seasonality. J. Hum. Evol. **120**, 203–214. (10.1016/j.jhevol.2017.08.011) 28870375

[73] Schulz E, Calandra I, Kaiser TM. 2010 Applying tribology to teeth of hoofed mammals. Scanning **32**, 162–182. (10.1002/sca.20181) 20949615

[74] Uno KT, Rivals F, Bibi F, Pante M, Njau J, de la Torre I. 2018 Large mammal diets and paleoecology across the Oldowan–Acheulean transition at Olduvai Gorge, Tanzania from stable isotope and tooth wear analyses. J. Hum. Evol. **120**, 76–91. (10.1016/j.jhevol.2018.01.002) 29752005

[75] Grine FE. 1986 Dental evidence for dietary differences in Australopithecus and Paranthropus: a quantitative analysis of permanent molar microwear. J. Hum. Evol. **15**, 783–822. (10.1016/s0047-2484(86)80010-0)

[76] Merceron G, Taylor S, Scott R, Chaimanee Y, Jaeger JJ. 2006 Dietary characterization of the hominoid Khoratpithecus (Miocene of Thailand): evidence from dental topographic and microwear texture analyses. Die Naturwissenschaften **93**, 329–333. (10.1007/s00114-006-0107-0) 16604335

[77] Scott RS, Ungar PS, Bergstrom TS, Brown CA, Grine FE, Teaford MF, Walker A. 2005 Dental microwear texture analysis shows within-species diet variability in fossil hominins. Nature **436**, 693–695. (10.1038/nature03822) 16079844

[78] Domning DP, Beatty BL. 2015 Use of tusks in feeding by dugongid sirenians: observations and tests of hypotheses. Anat. Rec. **290**, 523–538. (10.1002/ar.20540) 17516442

[79] Ford JKB, Ellis G, Matkin C, Wetklo M, Barrett-Lennard L, Withler R. 2011 Shark predation and tooth wear in a population of northeastern Pacific killer whales. Aquat. Biol. **11**, 213–224. (10.3354/ab00307)

[80] Thewissen JGM, Sensor JD, Clementz MT, Bajpai S. 2011 Evolution of dental wear and diet during the origin of whales. Paleobiology **37**, 655–669. (10.1666/10038.1)

[81] Fahlke JM, Bastl KA, Semprebon GM, Gingerich PD. 2013 Paleoecology of archaeocete whales throughout the Eocene: dietary adaptations revealed by microwear analysis. Palaeogeogr. Palaeoclimatol. Palaeoecol. **386**, 690–701. (10.1016/j.palaeo.2013.06.032)

[82] Lambert O, Martínez-Cáceres M, Bianucci G, Di Celma C, Salas-Gismondi R, Steurbaut E, Urbina M, de Muizon C. 2017 Earliest Mysticete from the Late Eocene of Peru sheds new light on the origin of baleen whales. Curr. Biol. **27**, 1535–1541.(10.1016/j.cub.2017.04.026) 28502655

[83] Bethune E, Schulz-Kornas E, Lehnert K, Siebert U, Kaiser TM. 2021 Tooth microwear texture in the eastern Atlantic harbour seals (Phoca vitulina vitulina) of the German Wadden Sea and Its implications for long term dietary and ecosystem changes. Front. Ecol. Evol. **9**. (10.3389/fevo.2021.644019)

[84] Marx FG, Hocking DP, Park T, Pollock TI, Parker WMG, Rule JP, Fitzgerald EMG, Evans AR. 2023 Suction causes novel tooth wear in marine mammals, with implications for feeding evolution in baleen whales. J. Mamm. Evol. **30**, 493–505. (10.1007/s10914-022-09645-1)

[85] Young MT, Brusatte SL, Beatty BL, De Andrade MB, Desojo JB. 2012 Dakosaurus. Anat. Rec. **295**, 1147–1158. (10.1002/ar.22491) 22577071

[86] Winkler DE, Schulz-Kornas E, Kaiser TM, Tütken T. 2019 Dental microwear texture reflects dietary tendencies in extant Lepidosauria despite their limited use of oral food processing. Proc. R. Soc. B **286**, 20190544. (10.1098/rspb.2019.0544) PMC6545078 31113323

[87] Schubert BW, Ungar PS. 2005 Wear facets and enamel spalling in tyrannosaurid dinosaurs. Acta Palaeontol. Pol. **50**. (10.1016/0043-1648(60)90132-0)

[88] Holwerda FM, Beatty BL, Schulp AS. 2013 Dental macro- and microwear in Carinodens belgicus , a small mosasaur from the type Maastrichtian. Neth. J. Geosci. Geol. En Mijnb. **92**, 267–274. (10.1017/s0016774600000202)

[89] Candeiro CRA, Currie PJ, Candeiro CL, Bergqvist LP. 2015 Tooth wear and microwear of theropods from the Late Maastrichtian Marília Formation (Bauru Group), Minas Gerais State, Brazil. Earth Environ. Sci. Trans. R. Soc. Edinb. **106**, 229–233. (10.1017/s175569101600013x)

[90] Varriale FJ. 2016 Dental microwear reveals mammal-like chewing in the neoceratopsian dinosaur Leptoceratops gracilis. PeerJ **4**, e2132. (10.7717/peerj.2132) 27441111 PMC4941762

[91] Calandra I. 2022 A workflow for quality control in surface texture analysis applied to teeth and tools. J. Archaeol. Sci. **46**, 103692. (10.1016/j.jasrep.2022.103692)

[92] Ungar PS, Brown CA, Bergstrom TS, Walkers A. 2003 Quantification of dental microwear by tandem scanning confocal microscopy and scale-sensitive fractal analyses. Scanning **25**, 185–193. (10.1002/sca.4950250405) 12926610

[93] Scott RS, Ungar PS, Bergstrom TS, Brown CA, Childs BE, Teaford MF, Walker A. 2006 Dental microwear texture analysis: technical considerations. J. Hum. Evol. **51**, 339–349. (10.1016/j.jhevol.2006.04.006) 16908052

[94] Herbst EC, Lautenschlager S, Bastiaans D, Miedema F, Scheyer TM. 2021 Modeling tooth enamel in FEA comparisons of skulls: comparing common simplifications with biologically realistic models. iScience **24**, 103182. (10.1016/j.isci.2021.103182) 34761178 PMC8567004

[95] Lautenschlager S, Figueirido B, Cashmore DD, Bendel EM, Stubbs TL. 2020 Morphological convergence obscures functional diversity in sabre-toothed carnivores. Proc. R. Soc. B **287**, 20201818. (10.1098/rspb.2020.1818) PMC7542828 32993469

[96] Ma W .S, Pittman M, Butler RJ, Lautenschlager S. 2022 Macroevolutionary trends in theropod dinosaur feeding mechanics. Curr. Biol. **32**, 677–686. (10.1016/j.cub.2021.11.060) 34919807

[97] Marcé-Nogué J. 2022 One step further in biomechanical models in palaeontology: a nonlinear finite element analysis review. PeerJ **10**, e13890. (10.7717/peerj.13890) 35966920 PMC9373974

[98] Ruiz JV, Ferreira GS, Lautenschlager S, de Castro MC, Montefeltro FC. 2023 Different, but the same: inferring the hunting behaviour of the hypercarnivorous bush dog (Speothos venaticus) through finite element analysis. J. Anat. **242**, 553–567. (10.1111/joa.13804) 36485003 PMC10008295

[99] Bright JA. 2014 A review of paleontological finite element models and their validity. J. Paleontol. **88**, 760–769. (10.1666/13-090)

[100] Palci A, LeBlanc ARH, Panagiotopoulou O, Cleuren SGC, Mehari Abraha H, Hutchinson MN, Evans AR, Caldwell MW, Lee MSY. 2021 Plicidentine and the repeated origins of snake venom fangs. Proc. R. Soc. B **288**, 20211391. (10.1098/rspb.2021.1391) PMC8354744 34375553

[101] Pollock TI, Panagiotopoulou O, Hocking DP, Evans AR. 2022 Taking a stab at modelling canine tooth biomechanics in mammalian carnivores with beam theory and finite-element analysis. R. Soc. Open Sci. **9**, 220701. (10.1098/rsos.220701) 36300139 PMC9579775

[102] Dumont ER, Grosse IR, Slater GJ. 2009 Requirements for comparing the performance of finite element models of biological structures. J. Theor. Biol. **256**, 96–103. (10.1016/j.jtbi.2008.08.017) 18834892

[103] Klug C, De Baets K, Kröger B, Bell MA, Korn D, Payne JL. 2015 Normal giants? Temporal and latitudinal shifts of Palaeozoic marine invertebrate gigantism and global change. Lethaia **48**, 267–288. (10.1111/let.12104)

[104] Boyajian G, Lutz T. 1992 Evolution of biological complexity and its relation to taxonomic longevity in the Ammonoidea. Geology **20**, 983–986. (10.1130/0091-7613(1992)0202.3.co;2)

[105] Lemanis R, Zlotnikov I. 2023 Fractal-like geometry as an evolutionary response to predation? Sci. Adv. **9**, eadh0480. (10.1126/sciadv.adh0480) 37494450 PMC10371019

[106] Hammer Ø, Harper DAT, Ryan PD. 2001 PAST: paleontological statistics software package for education and data analysis. Palaeontol. Electron. **4**, 9.

[107] Rasch LJ, Martin KJ, Cooper RL, Metscher BD, Underwood CJ, Fraser GJ. 2016 An ancient dental gene set governs development and continuous regeneration of teeth in sharks. Dev. Biol. **415**, 347–370. (10.1016/j.ydbio.2016.01.038) 26845577

[108] Fraser GJ, Standing A, Underwood C, Thiery AP. 2020 The dental lamina: an essential structure for perpetual tooth regeneration in sharks. Integr. Comp. Biol. **60**, 644–655. (10.1093/icb/icaa102) 32663287

[109] Newberry JS. 1889 The Paleozoic fishes of North America. Monogr. U.S. Geol. Surv. **16**. 10.5962/bhl.title.39658

[110] McCurry MR, Evans AR, Fitzgerald EMG, McHenry CR, Bevitt J, Pyenson ND. 2019 The repeated evolution of dental apicobasal ridges in aquatic-feeding mammals and reptiles. Biol. J. Linn. Soc. **127**, 245–259. (10.1093/biolinnean/blz025)

[111] Moller IJ, Melsen B, Jensen SJ, Kirkegaard E. 1975 A histological, chemical and X-ray diffraction study on contemporary (Carcharias glaucus) and fossilized (Macrota odontaspis) shark teeth. Arch. Oral Biol. **20**, 797–802. (10.1016/0003-9969(75)90056-4) 782408

[112] Hampe O, Long J. 1999 The histology of Middle Devonian chondrichthyan teeth from southern Victoria Land, Antarctica. Rec. West. Aust. Mus. **57**, 23–36.

[113] Gillis JA, Donoghue PCJ. 2007 The homology and phylogeny of chondrichthyan tooth enameloid. J. Morphol. **268**, 33–49. (10.1002/jmor.10501) 17146771

[114] Enault S, Guinot G, Koot MB, Cuny G. 2015 Chondrichthyan tooth enameloid: past, present, and future. Zool. J. Linn. Soc. **174**, 549–570. (10.1111/zoj.12244)

[115] Reif W. 1973 Morphologie und Ultrastruktur des Hai‐ ‘Schmelzes’. Zool. Scr. **2**, 231–250. (10.1111/j.1463-6409.1974.tb00753.x)

[116] Jambura PL, Pfaff C, Underwood CJ, Ward DJ, Kriwet J. 2018 Tooth mineralization and histology patterns in extinct and extant snaggletooth sharks, Hemipristis (Carcharhiniformes, Hemigaleidae)—Evolutionary significance or ecological adaptation? PLoS ONE **13**, e0200951. (10.1371/journal.pone.0200951) 30089138 PMC6082511

[117] Amini S, Razi H, Seidel R, Werner D, White WT, Weaver JC, Dean MN, Fratzl P. 2020 Shape-preserving erosion controlled by the graded microarchitecture of shark tooth enameloid. Nat. Commun. **11**, 5971. (10.1038/s41467-020-19739-0) 33235202 PMC7686312

[118] Martin RA, Hammerschlag N, Collier RS, Fallows C. 2005 Predatory behaviour of white sharks (Carcharodon carcharias) at Seal Island, South Africa. J. Mar. Biol. Assoc. UK **85**, 1121–1135. (10.1017/s002531540501218x)

[119] Chappell A, Séret B. 2021 Functional morphology of the feeding apparatus of the snaggletooth shark, Hemipristis elongata (Carcharhiniformes: Hemigaleidae). J. Anat. **238**, 288–307. (10.1111/joa.13313) 33107039 PMC7812129

[120] Scott JR. 2012 Dental microwear texture analysis of extant African Bovidae. Mammalia **76**, 157–174. (10.1515/mammalia-2011-0083)

[121] Scott RS, Teaford MF, Ungar PS. 2012 Dental microwear texture and anthropoid diets. Am. J. Phys. Anthropol. **147**, 551–579. (10.1002/ajpa.22007) 22331579

[122] Arman SD, Ungar PS, Brown CA, DeSantis LRG, Schmidt C, Prideaux GJ. 2016 Minimizing inter-microscope variability in dental microwear texture analysis. Surf. Topogr. **4**, 024007. (10.1088/2051-672X/4/2/024007)

[123] Jobbins M, Rücklin M, Sánchez Villagra MR, Lelièvre H, Grogan E, Szrek P, Klug C. 2024 Extreme lower jaw elongation in a placoderm reflects high disparity and modularity in early vertebrate evolution. R. Soc. Open Sci. **11**, 231747. (10.1098/rsos.231747) 38298398 PMC10827443

[124] Klug C, Kröger B, Korn D, Rücklin M, Schemm-Gregory M, de Baets K, Mapes RH. 2008 Ecological change during the early Emsian (Devonian) in the Tafilalt (Morocco), the origin of the Ammonoidea, and the first African Pyrgocystid Edrioasteroids, Machaerids and Phyllocarids. Palaeontogr. Abt. **283**, 83–176. (10.1127/pala/283/2008/83)

[125] Frey L, Rücklin M, Korn D, Klug C. 2018 Late Devonian and Early Carboniferous alpha diversity, ecospace occupation, vertebrate assemblages and bio-events of southeastern Morocco. Palaeogeogr. Palaeoclimatol. Palaeoecol. **496**, 1–17. (10.1016/j.palaeo.2017.12.028)

[126] Derycke C. 1992 Microrestes de Sélaciens et autres Vertébrés du Dévonien supérieur du Maroc. Bull. Mus. Natl. Hist. Nat., Sect. C, Sci. Terre, Paléontol., Géol., Minér. **14**, 15–61.

[127] Rücklin M, Clément G. 2017 Une révue des placodermes et sarcoptérygiens du Dévonien du Maroc. In Paléontologie des vertébrés du maroc: état des connaissances (ed. S Zouhri), pp. 79–102, vol. 180. Paris: Mémoires de la Société Géologique de France.

[128] Frey L, Pohle A, Rücklin M, Klug C. 2020 Fossil‐Lagerstätten, palaeoecology and preservation of invertebrates and vertebrates from the Devonian in the eastern Anti‐Atlas, Morocco. Lethaia **53**, 242–266. (10.1111/let.12354)

[129] Baets KD, Klug C, Korn D. 2011 Devonian pearls and ammonoid-endoparasite co-evolution. Acta Palaeontol. Pol. **56**, 159–180. (10.4202/app.2010.0044)

[130] Korn D. 1999 Famennian Ammonoid Stratigraphy of the Ma’der and Tafilalt (Eastern Anti-Atlas, Morocco). Abh. Geol. Bundesanst. **54**, 147–179.

[131] Korn D, Bockwinkel J. 2017 The genus Gonioclymenia (Ammonoidea; Late Devonian) in the Anti-Atlas of Morocco. Neues Jahrb. Für Geol. Und Paläontol. Abh. **285**, 97–115. (10.1127/njgpa/2017/0672)

[132] Becker R, Hartenfels S, Klug C, Dr. Aboussalam Z, Afhüppe L. 2018 The cephalopod-rich Famennian and Tournaisian of the Aguelmous Syncline (southern Maïder). Münst. Forsch. Zur Geol. Paläontol. **110**, 273–306.

[133] Hartenfels S, Becker RT. 2016 Age and correlation of the transgressive Gonioclymenia Limestone (Famennian, Tafilalt, eastern Anti-Atlas, Morocco). Geol. Mag. **155**, 1–44. (10.1017/S0016756816000893)

[134] Korn D, Hairapetian V, Gholamalian H. 2020 Gigantism in Late Devonian ammonoids from Chahriseh (Central Iran). Neues Jahrb. Für Geol. Und Paläontol. Abh. **297**, 287–294. (10.1127/njgpa/2020/0925)

[135] Sallan LC, Kammer TW, Ausich WI, Cook LA. 2011 Persistent predator–prey dynamics revealed by mass extinction. Proc. Natl Acad. Sci. USA **108**, 8335–8338. (10.1073/pnas.1100631108) 21536875 PMC3100987

[136] Korn D, Bockwinkel J, Ebbinghausen V, Walton SA. 2011 Beloceras, the most multilobate Late Devonian ammonoid. Bull. Geosci. **86**, 1–28. (10.3140/bull.geosci.1247)

[137] Van Valen L. 1973 A new evolutionary law. Evol Theory. In Foundations of macroecology (eds FA Smith, JL Gittleman, JH Brown), pp. 1–30, vol. 1. Chicago, Illinois 60637: University of Chicago Press. (10.7208/9780226115504)

[138] Klug C, Vallon LH. 2019 Regurgitated ammonoid remains from the latest Devonian of Morocco. Swiss J. Palaeontol. **138**, 87–97. (10.1007/s13358-018-0171-z)

[139] Greif M, Ferrón HG, Klug C. 2022 A new Meckel’s cartilage from the Devonian Hangenberg black shale in Morocco and its position in chondrichthyan jaw morphospace. PeerJ **10**, e14418. (10.7717/peerj.14418) 36573235 PMC9789696

[140] Greif M. 2025 Supplementary data for Greif et al. 2024 Reconstruction of feeding behaviour and diet in Devonian ctenacanth chondrichthyans using dental microwear texture and finite element analyses. Zenodo. (10.5281/zenodo.14605615) PMC11774596 39881788

[141] Calandra I, Greif M. 2025 Ivan-paleo/DMTA.Ctenacanths: For published paper. In Royal Society Open Science (v1.1, vol. 11, p. 240936). Zenodo. (10.5281/zenodo.14604218)

[142] Greif M, Calandra I, Lautenschlager S, Kaiser T, Mezane M, Klug C. 2024 Supplementary material from: Reconstruction of feeding behaviour and diet in Devonian ctenacanth chondrichthyans using dental microwear texture and finite element analyses. Figshare. (10.6084/m9.figshare.c.7590240) PMC11774596 39881788

